# Exosome-liposome hybrid nanoparticle codelivery of TP and miR497 conspicuously overcomes chemoresistant ovarian cancer

**DOI:** 10.1186/s12951-022-01264-5

**Published:** 2022-01-25

**Authors:** Longxia Li, Di He, Qianqian Guo, Zhiyoung Zhang, Dan Ru, Liting Wang, Ke Gong, Fangfang Liu, Yourong Duan, He Li

**Affiliations:** 1 Traditional Chinese Medicine Department, Renji Hospital, School of Medicine, Shanghai Jiao Tong University, Shanghai, 200127 China; 2 State Key Laboratory of Oncogenes and Related Genes, Shanghai Cancer Institute, Renji Hospital, School of Medicine, Shanghai Jiao Tong University, Shanghai, 200032 China; 3 Huashan Hospital and Key Laboratory of Medical Epigenetics and Metabolism and Molecular and Cell Biology Lab, Institute of Biomedical Sciences, Shanghai Medical College, Fudan University, Shanghai, 200032 China

**Keywords:** Anticancer therapy, Cisplatin-resistant ovarian cancer, TP, miR497, PI3K/AKT/mTOR

## Abstract

**Background:**

Although cisplatin-based chemotherapy has been used as the first-line treatment for ovarian cancer (OC), tumor cells develop resistance to cisplatin during treatment, causing poor prognosis in OC patients. Studies have demonstrated that overactivation of the phosphatidylinositol 3-kinase/protein kinase B/mammalian target of rapamycin (PI3K/AKT/mTOR) pathway is involved in tumor chemoresistance and that overexpression of microRNA-497 (miR497) may overcome OC chemotherapy resistance by inhibiting the mTOR pathway. However, the low transcriptional efficiency and unstable chemical properties of miR497 limit its clinical application. Additionally, triptolide (TP) was confirmed to possess a superior killing effect on cisplatin-resistant cell lines, partially through inhibiting the mTOR pathway. Even so, the clinical applications of TP are restricted by serious systemic toxicity and weak water solubility.

**Results:**

Herein, whether the combined application of miR497 and TP could further overcome OC chemoresistance by synergically suppressing the mTOR signaling pathway was investigated. Bioinspired hybrid nanoparticles formed by the fusion of CD47-expressing tumor exosomes and cRGD-modified liposomes (miR497/TP-HENPs) were prepared to codeliver miR497 and TP. In vitro results indicated that the nanoparticles were efficiently taken up by tumor cells, thus significantly enhancing tumor cell apoptosis. Similarly, the hybrid nanoparticles were effectively enriched in the tumor areas and exerted significant anticancer activity without any negative effects in vivo. Mechanistically, they promoted dephosphorylation of the overactivated PI3K/AKT/mTOR signaling pathway, boosted reactive oxygen species (ROS) generation and upregulated the polarization of macrophages from M2 to M1 macrophages.

**Conclusion:**

Overall, our findings may provide a translational strategy to overcome cisplatin-resistant OC and offer a potential solution for the treatment of other cisplatin-resistant tumors.

**Graphical Abstract:**

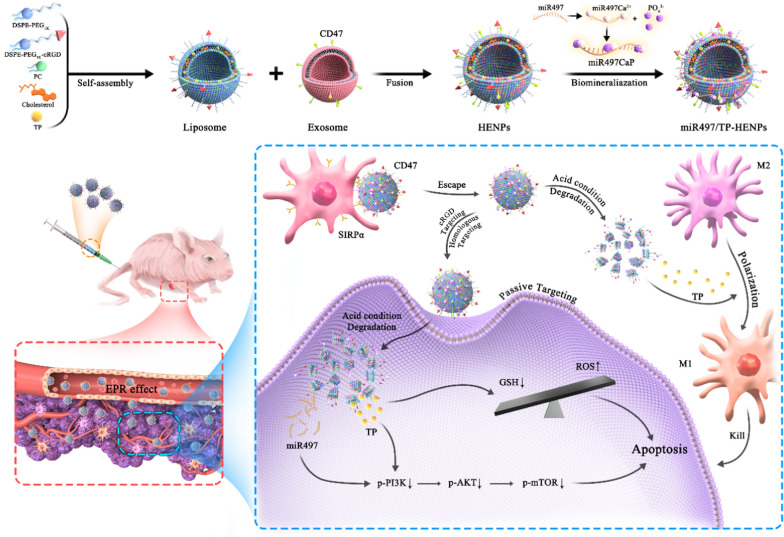

**Supplementary Information:**

The online version contains supplementary material available at 10.1186/s12951-022-01264-5.

## Background

Ovarian cancer (OC) is a highly lethal gynecologic tumor. It is also the second leading gynecologic malignancy among women, with approximately 13,770 deaths and nearly 21,410 new cases estimated for 2021 in the United States [1, 2]. The low survival rate for OC has been the case for decades [3]. Cisplatin increases survival in OC patients by interacting with DNA to form intrastrand crosslinking adducts and hence initiating proapoptotic signaling pathways [4–6]. However, chemical resistance has hampered its effectiveness in OC by various mechanisms involving reduced drug uptake, increased drug deactivation, enhanced DNA adduct repair, and activation of multiple signaling pathways that promote cell proliferation or suppression of pathways that promote cell death, such as loss of p53 function [7–9].

The clinical application of triptolide (TP) in drug-resistant OC is promising [10]. TP was extracted from the Chinese traditional medicine Tripterygium wilfordii Hook. F [11]. Several studies have demonstrated that TP suppresses the growth of chemotherapy-resistant cell lines in a variety of tumors, including pancreatic cancer, non-small cell carcinoma, breast cancer and OC [12–17]. TP also synergizes with multiple chemotherapeutic agents to overcome tumor resistance. For example, TP synergizes with cisplatin to overcome resistance in ovarian, lung, breast and bladder cancers, and TP is combined with paclitaxel to overcome resistance in prostate cancer and cervical carcinoma [18–20]. Overactivation of phosphatidylinositol 3-kinase/protein kinase B/mammalian target of rapamycin (PI3K/AKT/mTOR) signaling is associated with tumor-acquired chemotherapy resistance [21]. However, TP could effectively hinder the PI3K/AKT/mTOR signaling pathway [22, 23]. Moreover, an imbalance of glutathione (GSH) and reactive oxygen species (ROS) in OC cells may be responsible for drug resistance in OC [24, 25], and TP could correct the imbalance [26]. In addition, TP modulated the polarization of M2 macrophages to M1 macrophages to assist in the reversal of tumor resistance [27]. Compared with chemotherapeutic drugs targeting a single pathway, TP is involved in multiple antitumor pathways, and resistance to TP is less likely to be possessed. Therefore, TP represents a potential chemotherapeutic agent for cisplatin-resistant OC. Nevertheless, the severe systemic toxicity of TP and its poor water solubility resulting in low bioavailability limit its clinical application [28, 29].

Overexpressing microRNA-497 (miR497) may effectively overcome drug resistance in OC. Nevertheless, the low transcriptional efficiency and chemical instability of miR497 limit its utilization. miR497, a noncoding RNA of 22 nucleotides, belongs to one of the highly conserved miR-15/107 families [30]. miR497 exerts vital inhibitory effects on malignant tumors by restraining cell growth and eliciting apoptosis in lung cancer, hepatocellular carcinoma, osteosarcoma, and prostate cancer [31–33]. Studies have shown that miR497 can sensitize lung cancer cells to cisplatin resistance treatment in an AKT2-dependent manner [34]. Likewise, downmodulation of miR497 enhanced cell growth and cisplatin resistance in osteosarcoma by means of the PI3K/AKT pathway [35]. Xu’s results demonstrated that miR497 was downregulated in cisplatin-resistant OC, yet miR497 overexpression sensitized drug-resistant OC to cisplatin treatment by targeting mTOR/P70S6K1 [36]. However, most naked miRNAs are captured by endosomes, which causes poor intracellular delivery. Ultimately, inefficient gene interference occurs [37]. In vivo experiments usually use miRNA agomirs, which are specifically chemically modified to improve the stability of miRNAs and their ability to resist RNA enzymes [38].

Nanoplatform (NP)-mediated chemotherapy drug delivery has contributed to the development of clinical cancer therapy [39–42]. Liposomes are favored as popular drug delivery systems (DDSs) due to their self-assembly properties, ability to encapsulate water-soluble and lipophilic drugs and superior pharmacokinetic profile [43], and they deliver their encapsulated agents mainly through passive accumulation in specific tissues unless they carry additional surface ligands. The tumor-targeting peptide cRGD (cyclic arginine-glycine-aspartate acid) specifically targets the cell attachment receptor integrin α_v_β_3_ integrin, which is widely used as a target for tumor diagnosis and therapy because it is overexpressed in various tumor cells [44]. Liposomes are available for the active targeting of tumor cells by the modification of cRGD [45]. However, the property that liposomes are vulnerable to clearance by the mononuclear phagocyte system (MPS) restricts their cargo delivery application.

Exosomes have attracted substantial attention as DDSs due to their nanosized particles (30–150 nm), excellent biocompatibility, superior transcellular cross-communication, inherent hematological stability, low immunogenicity and homing targeting [46]. CD47 (a transmembrane protein) is highly expressed in tumor-derived exosomes [47]. The binding of CD47 and signal-regulatory protein alpha (SIRPα) serves as a “do not eat me” signal, thus evading phagocytosis by the MPS [48]. However, the inefficient drug encapsulation of exosomes is still an urgent challenge to be solved.

Recently, numerous studies have shown that hybrid nanoparticles, formed by membrane fusion of engineered exosomes (transfected with CD47 genes) and liposomes, increased drug delivery and avoided clearance by the MPS system [49]. The hybrid nanovesicles were characterized by excellent biocompatibility, prolonged circulation time, nonremoval by the MPS, and precise targeting to the tumor site for the rapid release of drugs [47, 50, 51].

**Fig. 1:**
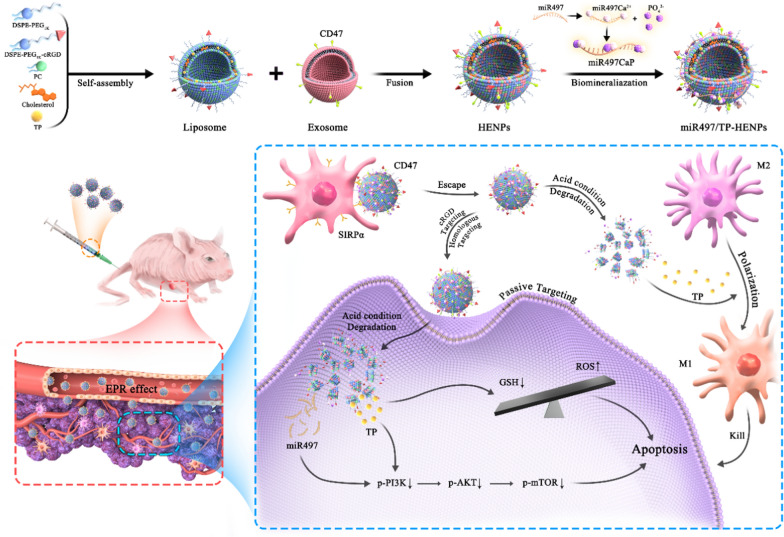
Diagram of the formative process and mechanism of action of miR497/TP-HENPs. miR497/TP-HENPs were synthesized by membrane fusion and biomineralization methods. First, liposomes were synthesized by assembly with DSPE-PEG_1k_-cRGD, phosphatidylcholine (PC), cholesterol and encapsulated TP. Then, liposomes and exosomes were fused by membrane fusion. Finally, CaP adsorbed miR497 on the surface of nanoparticles. The mechanisms by which miR497/TP-HENPs operated in OC cells were as follows: **a** the enhanced permeability and retention (EPR) effect were generated from the nanoscale size of the nanoplatforms, **b** the homing targeting of exosomes and cRGD further enhanced the targeting efficiency of nanoparticles, **c** CD47 on the exosome surface avoided nanoparticle clearance by the MPS system, **d** miR497 and TP synergistically inhibited the PI3K/AKT/mTOR pathway, **e** TP stimulated ROS production, and **f** TP modulated polarization of M2 macrophages into M1 macrophages, synergistically overcoming OC resistance

The purpose of this article was to verify whether the combination of miR497 and TP, targeting diverse regulatory mechanisms, effectively overcomes cisplatin-resistant OC. We designed a new bioinspired hybrid nanoplatform, namely, miR497/TP-HENPs, composed of exosomes from SKOV3-CDDP cells and liposomes modified by the target peptide cRGD, with the chemotherapeutic drug TP as the cargo and calcium phosphate (CaP) as a medium to adsorb miR497 on the surface of nanoparticles by electrostatic adsorption to reduce drug leakage. These hybrid nanoparticles effectively target tumor sites through the homologous targeting effect of tumor cell-derived exosomes and cRGD targeting. Under acidic conditions in the tumor microenvironment, the bioinspired nanoparticles rapidly cleave and release miR497 and TP, which synergistically induce OC cell apoptosis by inhibiting the PI3K/AKT/mTOR signaling pathway. TP also depletes GSH in tumor cells and elevates intracellular ROS to promote tumor cell death. Finally, TP overcomes drug resistance in OC by regulating macrophage polarization. In conclusion, the homotarget properties of nanoparticles can dramatically augment their ability to target and be retained at cancer sites, thereby improving the effectiveness of cancer therapy and overcoming chemoresistance (Fig. 1).

## Results and discussion

### Synthesis and characterization of bioinspired nanoplatforms

To improve the targeting efficiency of nanoparticles, increase the drug encapsulation ratio and reduce the toxic side effects of anticancer drugs, we prepared and characterized pH-sensitive biomimetic targeted hybrid nanoparticles named HENPs, which integrated exosomes and liposomes.

**Fig. 2:**
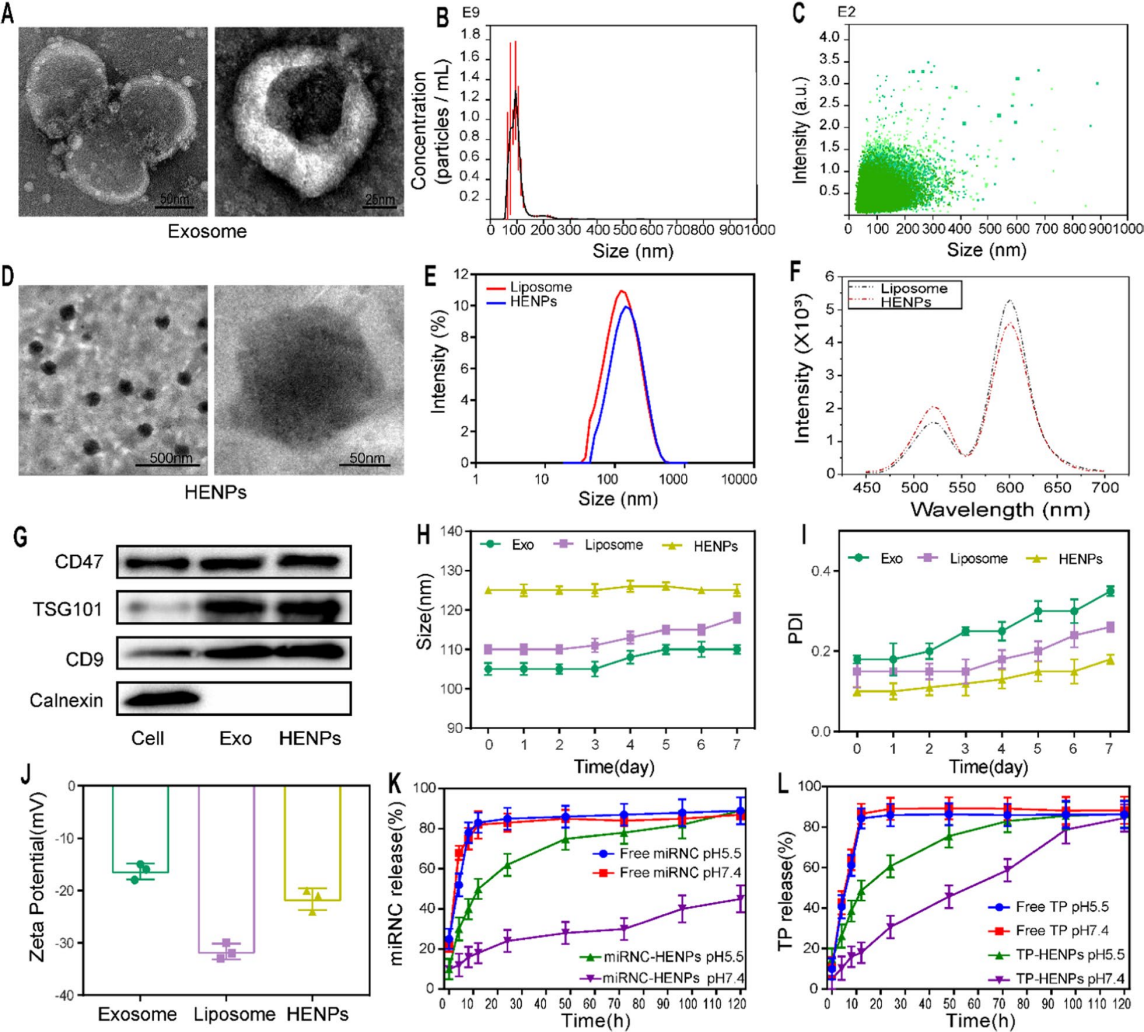
Synthesis and characterization of HENPs. **A** Representative image of exosomes captured by TEM at different magnifications. **B** The size distribution of exosomes and **C** the particle size distribution range of exosomes were measured by NTA. **D** Morphology of HENPs detected by TEM. **E** Size distribution of liposomes and HENPs. **F** The FRET assay showed the successful fusion of exosomes and liposomes. **G** Protein expression of exosomes and HENPs nanovesicles. **H** and **I** The nanoparticle size and PDI over a period of time were used to assess the stability of nanoparticles. **J** Zeta potential distribution of exosomes, liposomes and HENPs. **K** and **L** Release profiles of miRNC and TP at pH values of 5.5 and 7.4 at 37 °C

Tumor exosome-based nanoparticles are a promising and effective drug delivery platform [52]. In our study, exosomes were harvested from the conditioned culture supernatant of cisplatin-resistant SKOV3-CDDP cells according to classic ultracentrifugation as described in the methods [53]. The transmission electron microscopy (TEM) results displayed that the exosomes presented a typical cup-shaped morphology (Fig. 2A). The nanoparticle tracking analysis (NTA) results confirmed that the average particle size of the exosomes was approximately 104 ± 11 nm (Fig. 2B), and the distribution range of nanoparticles was 30–150 nm (Fig. 2C), suggesting that the exosomes we extracted conformed with the general standards.

The successful synthesis of cRGD and DSPE-PEG_1k_-COOH was verified by ^1^H-NMR (Additional file 1: Figure S1A, B). Liposomes were prepared by a simple thin film hydration technique and coupled with extrusion through a 200-nm polycarbonate membrane. TEM and DLS showed that the average particle size of spherical liposomes (Additional file 1: Figure S2) was 110 ± 15 nm (Fig. 2E).

Next, we hybridized exosomes and liposomes through ultrasound, membrane fusion and extrusion, constructing a biomimetic nanoparticle. The TEM images showed that the morphology of HENPs was uniform and round (Fig. 2D). The particle size of the hybrid nanoparticles was 125 ± 6 nm (Fig. 2E). The fusion efficiency of exosomes and liposomes was evaluated by fluorescence resonance energy transfer (FRET). The prehybridization spectra are shown as liposomes, while the posthybridization spectra are represented by HENPs spectra. We found that the peak emission of FITC at λem = 525 nm was elevated, but the peak emission of rhodamine B (RB) at λem = 595 nm was decreased after fusion occurred. The reduced FRET effect was attributed to the increased distance between both fluorescent dyes FITC and RB, indicating that the content of exosomes was inserted into the lipid bilayer of liposomes (Fig. 2F). To quantify the diminished FRET effect, we calculated the FRET efficiency. The results showed the quantification of FRET efficiency at different time. We found that the FRET efficiency decreased and finally remained at a relatively stable level over a period (Additional file 1: Figure S3). The above results demonstrated that the exosomes underwent membrane fusion with liposomes. Moreover, we used western blotting (WB) experiments to detect the characteristic proteins of exosomes to evaluate whether the components of exosomes changed during the fusion process. The results showed that the expression levels of CD47, TSG101 and CD9 in the exosome and HENPs groups were less different, while Calnexin was classified as a negative control (Fig. 2G). The structure of the exosomes did not change significantly in the HENPs.

All three nanoparticles were negatively charged (Fig. 2J), reducing nonspecific cellular uptake and prolonging circulation times due to electrostatic repulsion with negatively charged cell membrane surfaces. Subsequently, the stability of the three nanoparticles was evaluated by measuring the particle size and polydispersity index (PDI) after incubation in PBS with 10% fetal bovine serum (FBS) for 7 days. We found that compared with exosomes and liposomes, HENPs were more stable in the blood circulation because their particle size remained approximately 125 nm and the PDI was always less than 0.2. In contrast, the stability of exosomes was far less than that of HENPs (Fig. 2H, I). In vitro data revealed negligible toxicity of HENPs to OC cells, L929 fibroblasts and mouse macrophage RAW 264.7 cells (Additional file 1: Figure S4).

Then, we loaded TP and miR497 into the hybrid nanoparticles as detailed in the methods. A gel block test was performed to estimate the protective ability of miR497-HENPs to protect miR497 from RNase degradation. Free miR497 was quickly degraded by RNase, but no significant degradation of miR497-HENPs was observed after encapsulation for 48 h, suggesting that the nanoparticles protected miR497 from degradation by RNase (Additional file 1: Figure S5). We found that the encapsulation of the drug did not cause changes in the size and morphology of the nanoparticles. The hybrid nanoparticles had encapsulation efficiencies (EE%) of 78 ± 3% and 72 ± 5% for TP and miR497, respectively. Due to the pH-sensitive property of CaP, miR497/TP-HENPs disassembled intracellularly in an acidic environment, releasing the Cy5-miRNC and TP of HENPs more rapidly at a pH of 5.5 than at a pH of 7.4 (Fig. 2K, L). In contrast, there was no considerable impact of the pH level on the release of free Cy5-miRNC and TP, both of which reached 80% release within 20 h. These results revealed that miR497/TP-HENPs rapidly released the encapsulated drug in the acidic microenvironment rather than at normal sites.

In summary, we prepared miR497/TP-HENPs characterized by a suitable nanoparticle size, multitargeting capability, high drug encapsulation rate, valid drug protection and low clearance by MPS. These advantages of nanoparticles allowed forceful delivery of chemotherapeutic agents to the tumor site, ensuring a synergistic therapeutic effect of cisplatin-resistant OC.

### Uptake of HENPs by OC cells

**Fig. 3:**
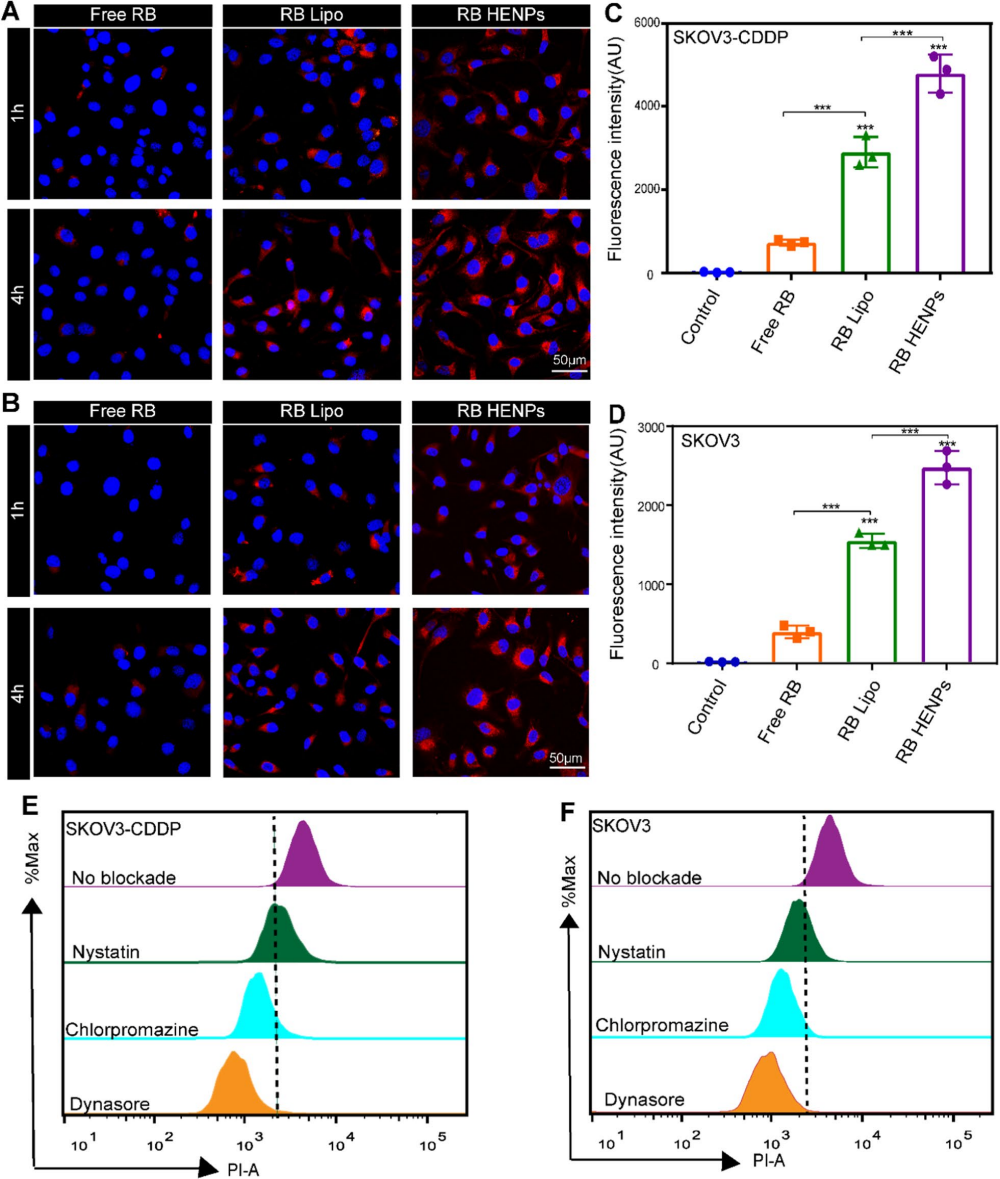
Cellular uptake of HENPs in vitro. CLSM was used to observe the cellular uptake of free RB or different nanoparticles encapsulated with RB in **A** SKOV3-CDDP and **B** SKOV3 cells (at a scale of 50 µm). **C**, **D** FCM was performed to analyze the cellular uptake of HENPs in SKOV3-CDDP and SKOV3 cells at 4 h. **E**, **F** RB HENPs cellular uptake mechanism determined by blocker. The data are expressed as the mean ± SD (ns: p > 0.05, *p < 0.05, **p < 0.01, ***p < 0.001)

In general, the superior antitumor effect requires effective cellular uptake of medicine by cancer cells. Therefore, the uptake ability of the hybrid nanoparticles by SKOV3-CDDP and SKOV3 cells was evaluated. First, we formulated nanoparticles loaded with a fluorescent dye, RB. Next, free RB, RB liposomes (RB Lipo) and RB HENPs were incubated with the two OC cell lines for different times. Finally, the results were visualized by confocal laser scanning microscopy (CLSM). We found that the fluorescence intensity was free RB < RB Lipo < RB HENPs (Fig. 3A, B), and the fluorescence intensity of RB was quantified (Additional file 1: Figure S6A, B), suggesting that compared to liposomes, biomimetic nanoparticles HENPs further increased the uptake of cargo by SKOV3-CDDP and SKOV3 cells in a time-dependent manner. Similarly, we observed the same results by flow cytometry (FCM), in which the mean fluorescence intensity (MFI) was significantly higher in the HENPs group than in the Lipo group and control group (Fig. 3C, D). It is worth mentioning that compared with SKOV3 cells, the fluorescence intensity in SKOV3-CDDP cells was always at a higher level, possibly due to the homing targeting properties of exosomes on maternal SKOV3-CDDP cells. The above results highlight that the hybridization of exosomes and liposomes improves the targeting ability of SKOV3-CDDP cells and the cellular uptake of drug-loaded nanovesicles.

To better understand the mechanism of the uptake of HENPs by OC cells, diverse internalization inhibitors were added to SKOV3-CDDP and SKOV3 cells to block specific uptake routes. We found that there was a modest difference between the nystatin (caveolae-mediated endocytosis inhibitor) group and the no-blockade group. However, the fluorescence intensity in SKOV3-CDDP and SKOV3 cells markedly decreased when incubated with chlorpromazine, a clathrin-dependent endocytosis inhibitor. Interestingly, dynasore, which simultaneously inhibited both uptake pathways mentioned above, exhibited the highest inhibition rate (Fig. 3E, F). The fluorescence intensity of RB was quantified (Additional file 1: Figure S6C, D). The above results suggest that the mechanism by which RB HENPs enter target cells is mainly mediated by clathrin-mediated endocytosis and assisted by caveolae-mediated endocytosis.

Nanoparticles were easily cleared by MPS systems, but CD47 on the exosome surface binding effectively to SIRPα serves as a “do not eat me” signal thus evading the clearance. Hence, CD47 was knocked down efficiently in SKOV3-CDDP cells to illustrate the function of CD 47 in avoiding clearance by MPS (Additional file 1: Figure S7A). We extracted exosomes derived from SKOV3-CDDP and SKOV3-CDDP_si-CD47_ cells. Two kinds of hybrid nanoparticles were synthesized, including LHENPs (hybrid nanoparticles with low expression of CD47) and HHENPs (hybrid nanoparticles with high expression of CD47). Then, both nanoparticles were labeled with the dye Dil. Fluorescence microscopy (FM) was employed to observe the cellular uptake of LHENPs and HHENPs at 4 h after incubation with RAW 264.7 cells. Fewer HHENPs were observed inside macrophages than LHENPs (Additional file 1: Figure S7B). These results suggest that the CD47 protein on the surface of exosomes derived from cancer cells can efficiently prevent nanoparticles from being phagocytosed by the MPS system.

### miR497/TP-HENPs overcome OC chemotherapy resistance by inhibiting the PI3K/AKT/mTOR signaling pathway in vitro

To evaluate the inhibitory effect of cisplatin and TP on OC cells, we performed a CCK-8 assay in two types of OC cells. The half-inhibitory concentrations (IC50) of cisplatin in two types of OC cells, SKOV3-CDDP and SKOV3, were 17.73 ± 2.58 µg mL^−1^ and 5.44 ± 1.37 µg mL^−1^, respectively, confirming an approximately threefold resistance to cisplatin in the SKOV3-CDDP cell line. TP exhibited higher antitumor potential than cisplatin in both cell lines, and there was no difference in the IC50 of TP (SKOV3-CDDP, 14.98 ± 1.92 ng mL^−1^ and SKOV3, 14.92 ± 2.52 ng mL^−1^) for either OC cell line at 48 h (Additional file 1: Figure S8A, B).

To investigate the potential anticancer effects of miR497/TP-HENPs in vitro, first, two OC cell lines were treated with different drugs at 24 h, 48 h and 72 h. We found that the cell viability of OC cells treated with miR497-HENPs, free TP, TP-HENPs, and miR497/TP-HENPs nanoplatforms was decreased in a time-dependent manner. The administration of miR497/TP-HENPs exhibited the highest cytotoxicity toward OC cells among all the groups (Additional file 1: Figure S9A, B), suggesting that miR497 and TP encapsulated in HENPs can synergistically overcome drug resistance in OC. Considering that nanoparticles encapsulating TP and miR497 are released more rapidly in an acidic environment, we further examined whether the antitumor effect of nanoparticles was further enhanced in an acidic environment. First, both OC cell lines were cultured with acidic medium (pH 5.5) and treated with PBS, miR497, miR497-HENPs, TP, TP-HENPs and miR497/TP-HENPs at 24 h, 48 h and 72 h. The absorbance values at 450 nm were measured after 1 h of CCK8 treatment. We found that the absorbance value in the blank group was approximately 0.5 at pH 5.5, while it was nearly 1.0 at pH7.4. These results indicated that the extremely acidic environment is toxic to OC cells in vitro. Therefore, direct observation of absorbance values provides a more accurate assessment of cell viability. Upon treating the cells with miR497, miR497-HENPs, TP, TP-HENPs and miR497/TP-HENPs, the absorbance values all showed a significant decline except for the miR497 group. We also discovered that the absorbance values of the TP, TP-HENPs and miR497/TP-HENPs groups were consistently lower than 0.4, and the miR497/TP-HENPs group inhibited tumor cell proliferation and exhibited maximum cellular toxicity (Additional file 1: Figure S10). Consequently, TP and miR497 were released rapidly under an acidic environment, showing a powerful antitumor effect in a shorter time period.

**Fig. 4:**
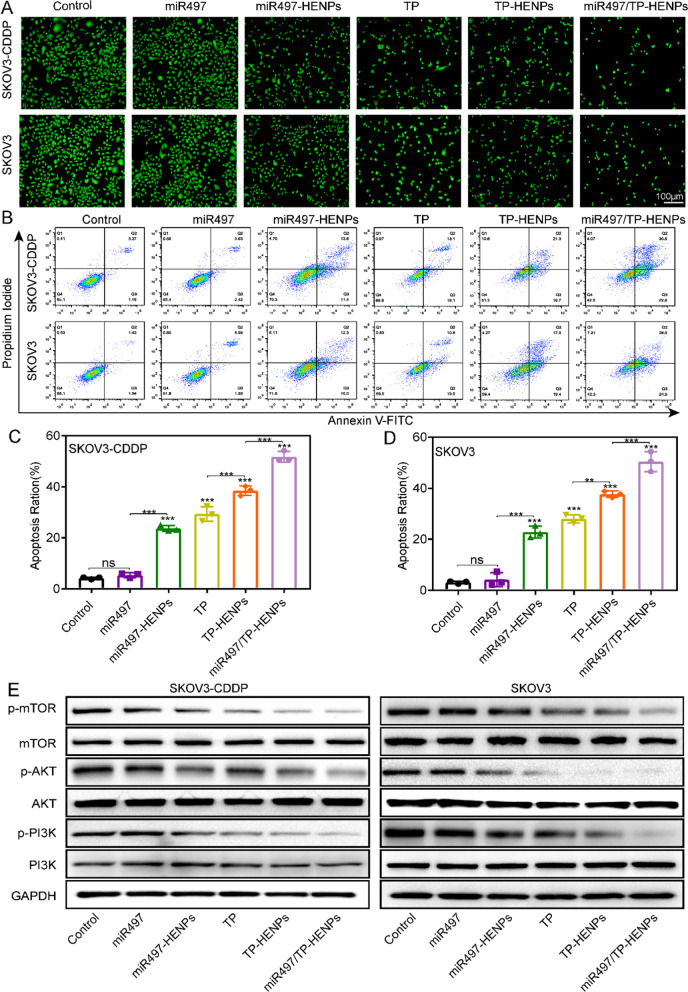
The combination of miR497and TP overcomes chemotherapy resistance in OC in vitro. **A** Images of the positive cells with calcein-AM staining. **B** Apoptotic SKOV3-CDDP and SKOV3 cells were analyzed by FCM after 48 h of treatment with different drugs in vitro. **C**, **D** Quantitative percentages of apoptotic cells in two ovarian cancer cell lines. **E** Western blotting images showed the expression levels of proteins of the PI3K/AKT/mTOR signaling pathway. The data are expressed as the mean ± SD (ns: p > 0.05, *p < 0.05, **p < 0.01, ***p < 0.001)

Next, the calcein AM staining test was performed to assess the viability of SKOV3-CDDP and SKOV3 cells. Obviously, green fluorescence (lives cells) in the control group and free miR497 group were both superbright, indicating that miR497 alone will not damage cells in vitro. Although treatment with miR497-HENPs caused some cell death, the least obvious green fluorescence was found in the miR497/TP-HENPs group (Fig. 4A), and the fluorescence intensity was quantified (Additional file 1: Figure S11A, B), consistent with the results of the CCK-8 assay.

Finally, the therapeutic efficiency of miR497/TP-HENPs was further confirmed by the Annexin V FITC/PI double staining assay. As shown in Fig. 4B and quantified in Fig. 4C and D, the cell apoptosis ratios of both types of OC cells treated with miR497/TP-HENPs (SKOV3-CDDP, 51.8 ± 1.7% and SKOV3, 50.5 ± 3.2%) were significantly higher than those of OC cells treated with the TP-HENPs (SKOV3-CDDP, 38.5 ± 1.5% and SKOV3, 37.7 ± 1.1%) and the miR497-HENPs (SKOV3-CDDP, 23.7 ± 1.0% and SKOV3, 22.8 ± 1.9%).

Overactivation of PI3K/AKT/mTOR signaling may cause tumors to acquire chemotherapy resistance [21, 54]. In approximately three-quarters of epithelial OCs, the mTOR pathway is hyperactivated, leading to tumorigenesis and chemoresistance [55]. Therefore, whether miR497/TP-HENPs overcome OC drug resistance mediated by the inhibition of the PI3K/AKT/mTOR signaling pathway was investigated. As illustrated in Fig. 4E, dephosphorylation of p-PI3K, p-AKT and p-mTOR protein was detected in these experimental groups, including SKOV3-CDDP and SKOV3 cells treated with miR497-HENPs, free TP, TP-HENPs and miR497/TP-HENPs but not in the PBS and free miR497 groups. The above results indicate that hybrid exosomes and liposomes and the combination of miR497 and TP improved the cellular uptake of drug-loaded nanovesicles and exerted anticancer effects by downregulating the PI3K/AKT/mTOR signaling pathway.

### miR497/TP-HENPs boost intracellular ROS and induce M2 to M1 polarization of macrophages

**Fig. 5:**
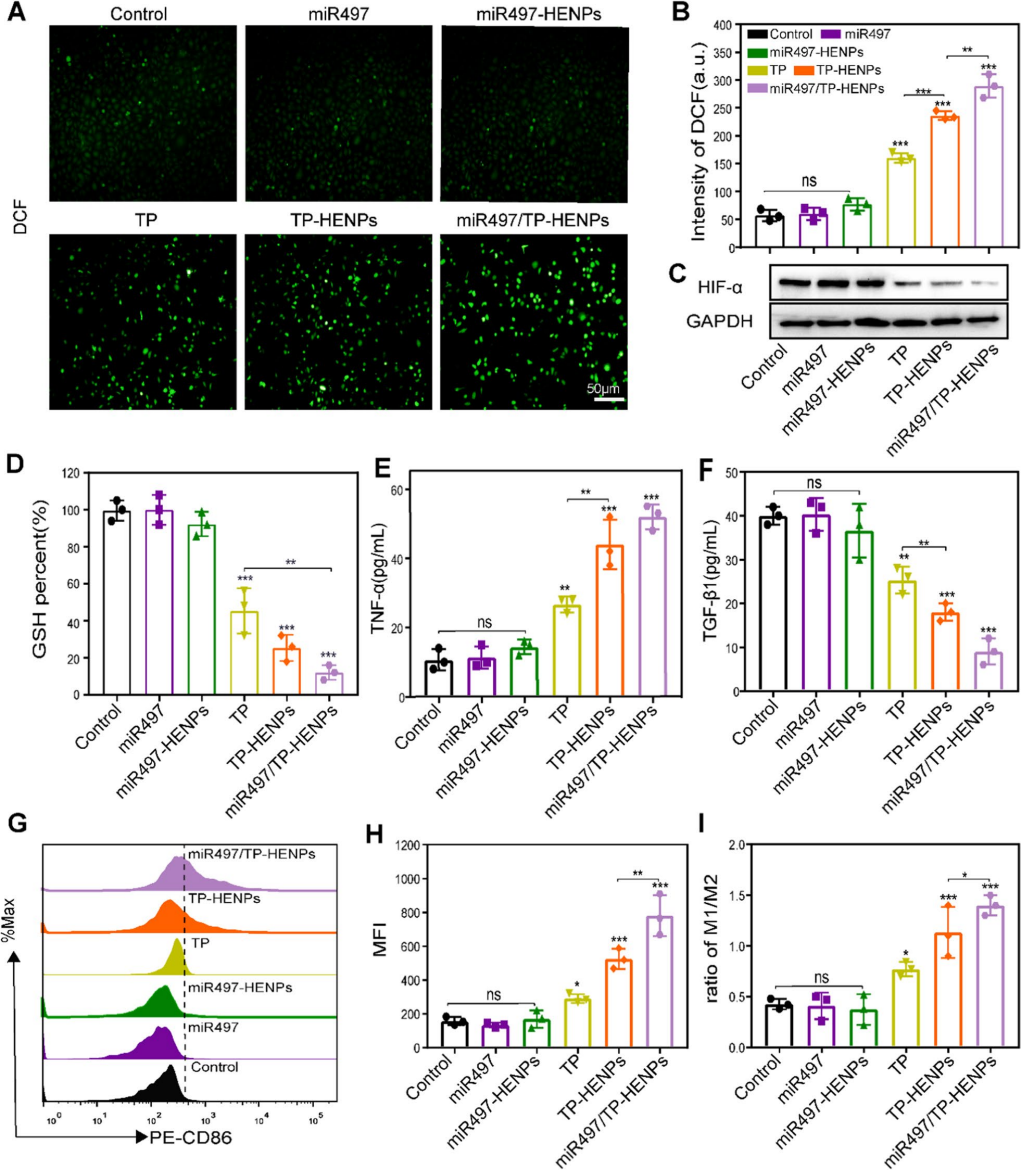
miR497/TP-HENPs boost intracellular ROS and induce M2 to M1 polarization of macrophages. **A** ROS in SKOV3-CDDP cells. Scale bar: 50 μm. **B** Quantitative analysis of ROS in SKOV3-CDDP cells by FCM. **C** Analysis of the expression of HIF-α using WB assay. **D** GSH quantity in SKOV3-CDDP cells. **E**, **F** Cell supernatant levels of TNF-α and TGF-β1 in different treatment groups of RAW264.7 macrophages. **G**, **H** FCM analysis of the expression of M1 macrophage markers (F4/80 + CD86 +) and quantitative analysis of the MFI of PE-CD86. **I** The ratio of M1/M2 subtype macrophages treated with different groups in vitro

TP promoted OC cell apoptosis by inducing the significant production of ROS. Zhang’s study showed that cisplatin-resistant OC cells tended to maintain high levels of GSH regardless of ROS reduction [56], suggesting that increased GSH may be related to the chemoresistance of tumors [57]. Therefore, boosting ROS generation and consuming GSH is a potential treatment strategy for drug-resistant OC. First, SKOV3-CDDP cells were treated with PBS, miR497, miR497-HENPs, TP, TP-HENPs and miR497/TP-HENPs. Then, the ROS detector 2,7-dichlorodihydrofluorescein (DCF) was used to assess the production of ROS by measuring the fluorescence intensity. The fluorescence intensity of miR497/TP-HENPs was the highest among all treatment groups, including improved fluorescence intensity of TP and TP-HENPs. However, the fluorescence intensities of miR497 and miR497-HENPs were basically not different from that of the control group (Fig. 5A, B). Next, we measured the hypoxia-inducible factor α (HIF-α) protein by WB assay to explore whether the nanoparticles could alleviate the effects of hypoxia. We found that the expression of HIF-α was significantly downregulated in the miR497/TP-HENPs group (Fig. 5C). Finally, the results of the intracellular GSH showed no difference in the miR497 and miR497-HENPs groups compared with the control group. It is worth mentioning that the miR497/TP-HENPs group had the lowest level of GSH (12% of the control group) compared with the TP and TP-HENPs groups (46% and 26% of the control group, respectively) in SKOV3-CDDP (Fig. 5D).

Tumor-associated macrophages (TAMs) have a significant role in cancer growth, progression, metastasis and angiogenesis. M1 macrophage polarization is a prerequisite for macrophage-based antitumor activity [58]. We induced RAW264.7 macrophages to differentiate into M2 macrophages by interleukin-4 (IL-4), treated them with different drugs, and then observed the regulatory effects of miR497/TP-HENPs on macrophages. ELISA was used to assess the changes in tumor necrosis factor-α (TNF-α) and transforming growth factor-β1 (TGF-β1) in cell supernatants. The results revealed that TNF-α was upregulated fivefold and TGF-β1 was decreased fourfold in the miR497/TP-HENPs group compared with the control group (Fig. 5E, F). Having investigated the polarization of M2 macrophages to M1 macrophages by FCM, we found that miR497/TP-HENPs promoted M1 macrophages most significantly, where the ratio of M1/M2 macrophages was 1.5 and that of the control group was 0.4 (Fig. 5G–I).

These above results implied that miR497 alone has no influence on the balance of ROS and GSH or conversion of macrophage phenotypes unless combined with TP. Therefore, we speculate that miR497 may enhance the antitumor effect of TP. In summary, miR497/TP-HENPs effectively induced tumor cell death by rebalancing intracellular GSH and ROS in tumor cells and upregulating M1 macrophage polarization, thus providing two adjuvant pathways to overcome OC drug resistance.

### miR497/TP-HENPs effectively inhibit the growth of cisplatin-resistant OC subcutaneous tumors in vivo

**Fig. 6:**
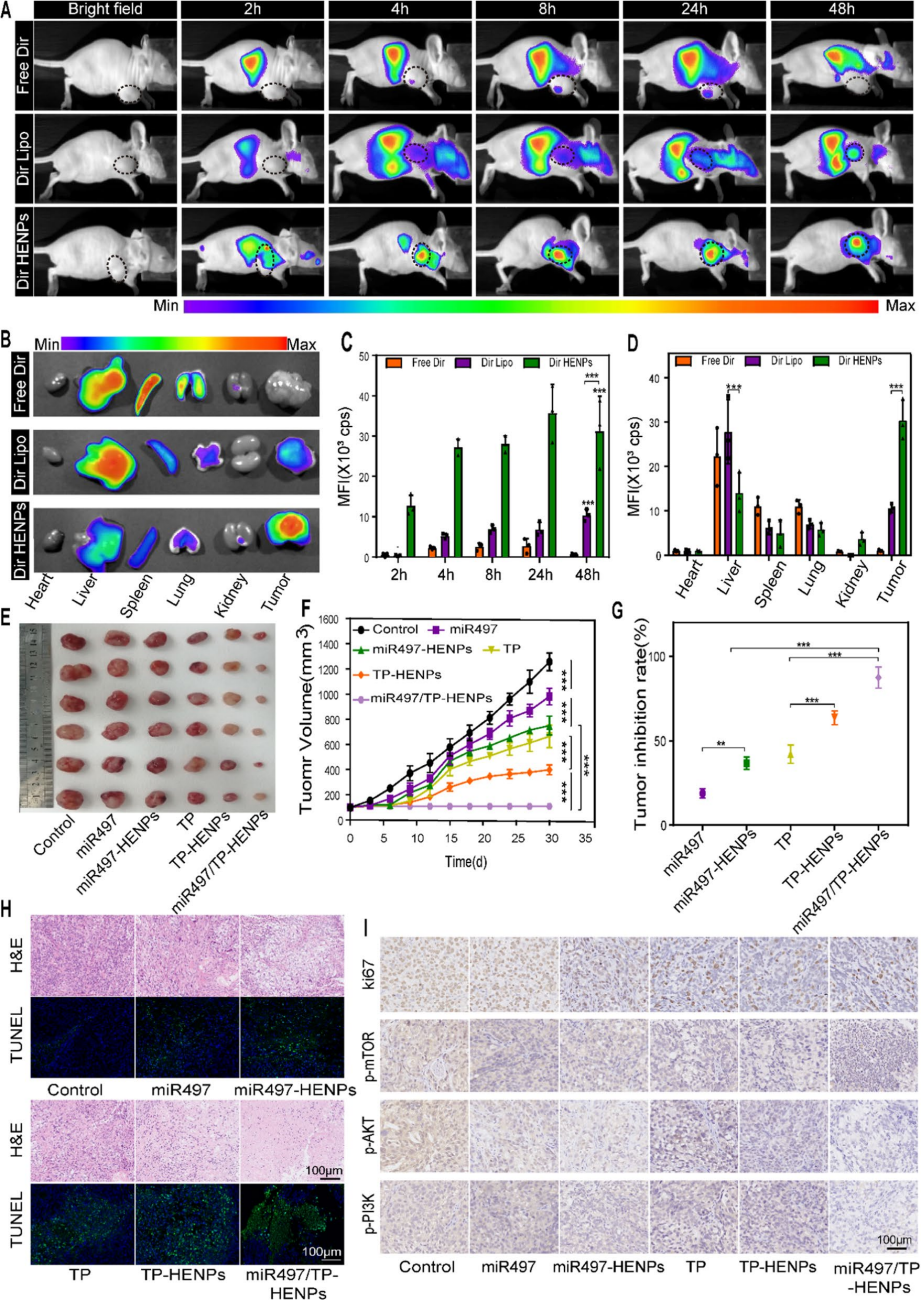
The targeting and antitumor activity of miR497/TP-HENPs in vivo. **A** In vivo imaging to observe the tumor targeting ability of different nanoparticles. **B** Ex vivo fluorescence images of the main organs and tumors isolated from mice bearing subcutaneous SKOV3-CDDP tumors. **C** Quantitative analysis of Dir distribution in the tumor site postinjection elevated by the fluorescence intensity measured in (**A**). **D** Quantitative assessment of the mean fluorescence intensity in major organs and isolated subcutaneous tumors. **E** Representative photographs of subcutaneous tumors harvested from all treatment groups. **F** Growth record curves of subcutaneous tumors in nude mice during the experiment. **G** The inhibition rate of OC treated with various drugs. **H** The H&E staining and TUNEL staining. **I** Immunohistochemical detection of ki67, p-PI3K, p-AKT, and p-mTOR. Scare bar: 100 µm

To evaluate the therapeutic potential of miR497/TP-HENPs in cisplatin-resistant OC in vivo, we established BALB/c-nu mice bearing subcutaneous SKOV3-CDDP tumors. In vitro experiments demonstrated that HENPs could target tumor cells, and thus, whether prepared nanoparticles have excellent targeting ability in vivo was investigated. An in vivo imaging apparatus was used to evaluate the tumor-targeting effect of HENPs in vivo. The free fluorescent dyes Dir, Dir liposomes (Dir Lipo) and Dir HENPs were intravenously injected into tumor-bearing nude mice and imaged by in vivo imaging at different times. We observed strong Dir fluorescence in the tumor sites but less Dir fluorescence in normal sites after administering Dir HENPs at 4 h, which lasted for 48 h. However, in both the free Dir group and Dir Lipo group, fluorescence accumulated in normal organs, and little fluorescence was detected in the tumor sites (Fig. 6A, C). Afterward, the mice were euthanized; subcutaneous tumors, hearts, livers, spleens, kidneys and lungs were harvested; and the intensity of Dir was measured. The fluorescence distributions in organs revealed a marked reduction in the hepatic and splenic accumulation of HENPs. HENPs exhibited better tumor accumulation than Dir Lipo and free Dir based on qualitative and quantitative data (Fig. 6B, D). These results showed that the majority of Dir HENPs were taken up by OC cells, whereas normal tissues showed minimal Dir HENPs uptake. These results presented a potent argument for miR497/TP-HENPs to exert antitumor effects in vivo.

In vivo experiments usually use miRNA agomirs, which are specifically chemically modified to improve the stability of miRNAs and their ability to resist RNA enzymes. However, we prepared hybrid nanoparticles HENPs characterized by multitargeting capability, valid miR497 protection and low clearance by MPS. These advantages of nanoparticles allowed specific delivery of miR497 to the tumor site. In vivo experiments, we chose the miR497 agomir in miR497 group and naked miR497 in miR497-HENPs group and miR497/TP-HENPs groups. We investigated the antitumor efficacy of miR497 and TP coencapsulated by HENPs in vivo. First, in line with our in vitro experiments, the tumors were smaller in size in all treatment groups than in the control group (Fig. 6E). The tumor volume was the smallest (average volume of 107 ± 27 mm^3^) and the tumor suppression rate (87%) was the highest in the miR497/TP-HENPs group. These results indicate that the codelivery of miR497 and TP exhibited a superior antitumor effect (Fig. 6F, G). In the histological analysis, hematoxylin and eosin (H&E) staining results showed that the maximal quantity of apoptotic cells in cisplatin-resistant OC was present in the miR497/TP-HENPs group. Moreover, terminal deoxynucleotidyl transferase dUTP nick-end labeling (TUNEL) staining displayed a significantly increased number of green fluorescence-positive apoptotic cells in the miR497/TP-HENPs group (Fig. 6H). We explored the antitumor mechanism of the observed effects. First, immunohistochemical (IHC) staining results confirmed that the miR497/TP-HENPs group had decreased expression of the proliferation-related protein ki67. Activation of the PI3K/AKT/mTOR signaling pathway was also examined by IHC, and the findings were consistent with the results of the in vitro experiments. Each drug treatment group exhibited a distinct extent of signaling pathway inhibition, with the miR497/TP-HENPs group showing the strongest level of suppression (Fig. 6I).

**Fig. 7:**
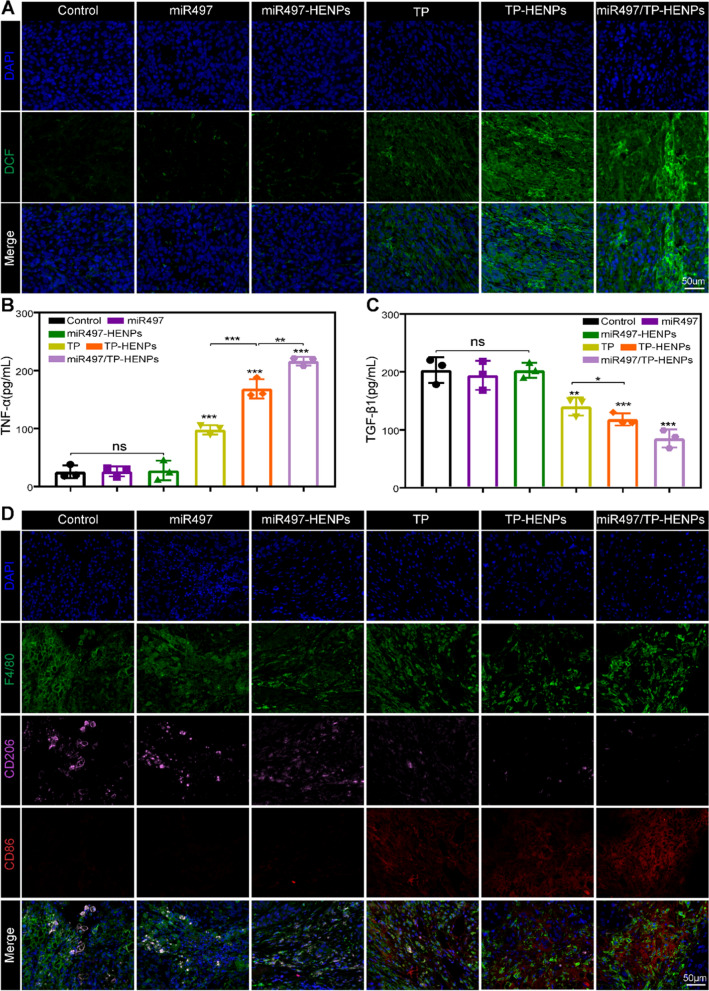
miR497/TP-HENPs induced ROS production in cisplatin-resistant OC and regulated macrophage polarization from M2 to M1 in vivo. **A** The ROS of tumor sections after treatments. Scale bar: 50 µm. **B**, **C** Serum levels of TNF-α and TGF-β1 in different treatment groups in vivo. One-way ANOVA was used to determine significant differences; *p < 0.05; **p < 0.01, ***p < 0.001, compared with the indicated groups. **D** Immunofluorescence staining images of different macrophage markers (M1, F4/80 + CD86 + and M2, F4/80 + CD206 +) in tumor tissue sections. Scale bar: 50 µm

Next, we assessed the level of ROS in tumor tissues. The results in Fig. 7A show that treatment with miR497/TP-HENPs caused the most obvious green fluorescence of DCF (high ROS). We also observed that miR497 alone did not induce a significant change in DCF (low ROS) compared with the control group. However, once miR497 was combined with TP, green fluorescence was clearly enhanced, and these results were in agreement with our in vitro experiments. Finally, we investigated the activation of the intrinsic immune system by detecting changes in the cytokines TNF-α and TGF-β1 in blood samples and assessing the polarization of macrophages. We observed that TP elevated TNF-α while decreasing TGF-β1. Moreover, HENPs-encapsulated TP further promoted the above effects, which were even more pronounced after combination with miR497 (Fig. 7B, C). Regarding macrophage polarization, immunofluorescence showed a significant number of M2 macrophages (F4/80 + CD206 +) and almost no M1 macrophages (F4/80 + CD86 +) in the control, miR497 and miR497-HENPs groups. However, the opposite results were observed in the TP-containing treatment group. The ratios of M1 macrophages to M2 macrophages in mice treated with TP-HENPs were higher than those in mice treated with free TP. Remarkably, the miR497/TP-HENPs group had the highest number of M1 macrophages compared with the other treatments. These results showed that TP upregulated the polarization of M1 macrophages, and the polarization effect was enhanced when miR497 was combined, which due to that miR497 may enhance the antitumor properties of TP (Fig. 7D).

The remarkable ability to target tumors ensured that the therapeutic effect of the biointelligent HENPs group was superior to that of the free drug group. Moreover, the miR497/TP-HENPs group showed the most pronounced inhibition of tumor growth, indicating that coloaded chemotherapeutic agents improve antitumor efficiency. In summary, the in vivo experiments showed that miR497/TP-HENPs effectively overcame drug resistance in OC.

### Safety of miR497/TP-HENPs in vivo

**Fig. 8:**
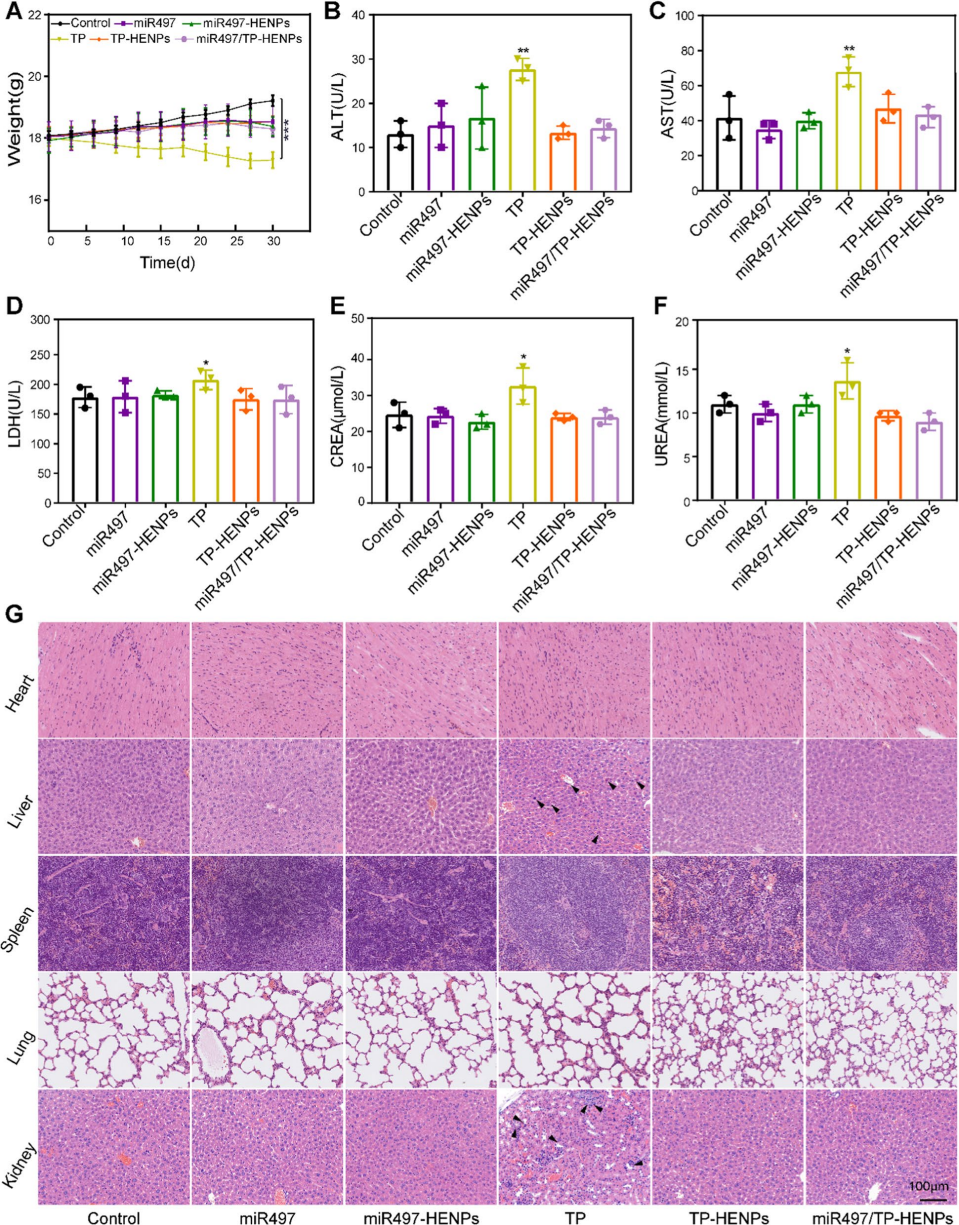
In vivo safety assessment. **A** Body weight change curves of nude mice in all treatment groups during the trial. **B-F** Changes in biochemical indicators of liver and kidney function in mice after the last injection. All data were from three replicates. **G** H&E staining of the main organs of nude mice and the structural damage sites were noted with black arrows. Scale bar: 100 μm

To assess the safety of the miR497/TP-HENPs nanoplatform in vivo, we measured and recorded the changes in mouse body weight in all treatment groups throughout the entire trial. The body weight of mice in most groups exhibited no significant change (average body weight, 18.4 ± 0.07 g), except for those of the free TP group (17.3 ± 0.22 g) and the control group (19.6 ± 0.42 g). The decrease in mouse body weight in the free TP group contributed to the systemic toxicity of free TP, while uncontrollable growth of tumors in the control group resulted in an increase in mouse body weight (Fig. 8A). Furthermore, we also found that the levels of ALT, AST, LDH, CREA and UREA of all treatment groups were at a lower level, but those of the free TP group were all at a higher level (Fig. 8B–F), indicating the hepatotoxicity and nephrotoxicity of free TP. In addition, we further analyzed the histomorphological changes in the main organs of the tumor-bearing mice in various treatment groups. Similarly, only the free TP group showed liver and kidney damage, whereas all other groups did not display tissue injury (Fig. 8G). These results confirmed that HENPs have the ability to reduce the side effects of free drugs in normal organs via less toxic delivery materials.

## Conclusion

The extreme susceptibility of OC cells to develop resistance to the chemotherapeutic drug cisplatin remains a formidable obstacle in OC treatment. miR497 and TP are promising in overcoming drug resistance in OC. However, the application of these two agents was hindered by the low transcriptional efficiency of miR497 and serious systemic toxicity and weak water solubility of TP. To solve the above dilemma, we synthesized hybrid nanoparticles to deliver miR497 and TP. Ideal drug delivery systems (DDSs) are characterized by excellent biocompatibility, prolonged circulation in the blood, nonremoval by the mononuclear phagocyte system (MPS) and precise targeting of the tumor site for the rapid release of drugs [43]. According to the present study, the low loading rate, toxicity and induced immune response of nanocarriers still hinder the further development of DDSs [59].

In our study, we constructed bioinspired hybrid nanoparticles named miR497/TP-HENPs that fused liposomes and exosomes and coencapsulated both chemotherapy agents TP and miR497. The designed nanoparticles successfully overcome the chemotherapeutic resistance of OC in vitro and in vivo. miR497/TP-HENPs are characterized by a nanoparticle size, high drug encapsulation efficiency and nucleic acid protection and are capable of existing stably and continuously releasing TP and miR497. The appropriate particle size and dual active targeting, including homologous targeting and cRGD targeting, ensure effective accumulation of the hybrid miR497/TP-HENPs at tumor sites. It is worth emphasizing that hybrid nanocarriers specifically block the PI3K/AKT/mTOR signaling pathways in OC cells. Additionally, they can reduce the level of intracellular GSH and increase ROS, thereby destroying the normal cell microenvironment and causing tumor cell death. Finally, the nanoparticles upregulate M1 macrophage polarization to inhibit OC progression. In general, TP, miR497, ROS and M1 macrophages all work together to overcome drug resistance in OC. This hybrid nanoparticle has a wide range of uses and very low toxicity to normal tissues.

Our findings reinforce the concept that combining TP with miR497 achieves better clinical effects, and nanoparticle encapsulation tackles the dilemma of chemotherapy drugs. We believe that this work will be a promising candidate to overcome cisplatin resistance in OC.

## Experimental section

### Materials and methods

#### Materials and cells

1,2-Distearoyl-sn-glycero-3-phosphoethanolamine-N-carboxy (polyethylene glycol)-1000 (DSPE-PEG_1k_-COOH) and cRGD peptide were purchased from Meiluo Technology (Shenzhen, China). Phosphatidylcholine (PC) and cholesterol were acquired from Ponsure Biotechnology (Shanghai, China). Cisplatin, calcium chloride (CaCl_2_) and 4-(2-hydroxyethyl)-1-piperazineetha-nesulfonic acid (HEPES) were obtained from Sigma–Aldrich. Triptolide (purity > 99.8%) was obtained from Chengdu Must Biotechnology (Sichuan, China). miR497–5p (5’-CAGCAGCACACUGUGGUUUGU-3’), micrONTM mimic Negative Control #22 and Cy5-miRNA (Cy5-miRNC) were purchased from Ribo-Bio Biotechnology (Guangzhou, China). 4,6-Diamidino-2-phenylindole (DAPI) and Hoechst 33,342 were obtained from the Beyotime Institute of Biotechnology (Jiangsu, China). Zombie-apc-cy7, anti-F4/80-BV421, anti-CD86-PE and anti-CD206-APC were purchased from BioLegend (San Diego, USA). HRP-conjugated secondary antibodies and FITC were purchased from Servicebio Technology (Wuhan, China). ELISA kits for tumor necrosis factor-α (TNF-α) and transforming growth factor-β1 (TGF-β1) were purchased from Multisciences (Zhejiang, China). Trypsin − EDTA (0.25%), Dulbecco’s modified Eagle’s medium (DMEM), fetal bovine serum (FBS) and penicillin–streptomycin were obtained from Gibco (USA).

The human ovarian cancer cell line SKOV3, cisplatin-resistant human ovarian cancer cell line SKOV3-CDDP, mouse fibroblast cell line L929 and mouse macrophage cell line RAW264.7 were provided by the State Key Laboratory of Oncogenes and Related Genes (Shanghai, China). All cells were cultured in DMEM supplemented with 10% FBS and 1% penicillin–streptomycin at 37 °C with 5% (v/v) CO_2_ in a humidified atmosphere.

#### Gene silencing of CD47 in vitro

SKOV3-CDDP cells were plated in 6-well culture plates and cultured overnight. Then, cells were transfected with negative control siRNA as the blank group or CD47 siRNA as the experimental group according to the manufacturer’s protocol (Ribo-Bio). The gene silencing effect of siRNA was quantitated by real-time PCR.

#### Extraction and purification of exosomes

Exosomes were isolated and purified according to our previous study, and the details are as follows. SKOV3-CDDP or SKOV3-CDDP_si-CD47_ cells were cultured in DMEM with 10% exosome-free (SBI) bovine serum for 48 h. Then, the cell supernatant was collected and centrifuged at 300 g for 10 min, 2000 g for 10 min and 10,000 g for 30 min to remove residual live cells, dead cells and cell debris, respectively. Next, the supernatant was collected and centrifuged at 100,000 g for 70 min at 4 °C to precipitate the exosomes. To purify the exosomes, the supernatant was washed with PBS and then centrifuged at 100,000 g for 70 min at 4 °C. Ultrafiltration was performed at 12,000 g for 30 min at 4 °C, followed by filtration through a 0.22 μm filter to concentrate the exosomes. PBS-resuspended exosomes were used immediately or stored at − 80 °C.

#### Synthesis of DSPE-PEG_1k_-cRGD

Twenty-five milligrams of COOH-PEG_1k_-DSPE, 3 mg of N-hydroxysuccinimide (NHS) (2 eq) and 5.4 mg of 1-ethyl-3-(3-dimethylaminopropyl) carbodiimide hydrochloride (EDC) (2 eq) were dissolved in a 2 ml mixture of methanol and chloroform (V/V = 1:1) in a round-bottomed flask, followed by stirring for 4 h at room temperature. Next, a mixture of 1 mL of deionized water containing 8.2 mg of cRGD (1 eq) peptide and 5 mL of methanol was added, and the reaction continued for 6 h. Then, all solutions were removed by spin evaporation, and the obtained solid was dialyzed in a 500 molecular retention capacity dialysis bag for 3 days. Finally, the dialysate was lyophilized to obtain DSPE-PEG_1k_-cRGD. The construction of DSPE-PEG_1k_-cRGD was confirmed by ^1^H NMR (500 MHz) spectroscopy, with CDCl_3_ as the solvent.

#### Preparation of liposomes

Liposomes were synthesized according to the thin film method. First, PC/DSPE-PEG_1k_-cRGD/cholesterol was mixed in dichloromethane at a molar ratio of 45:5:2 (mol/mol/mol) [49, 60], vortexed quickly and completely dissolved. Next, 1 mL of DEPC water was added and ultrasonicated for 3 min at 4 °C (33% amplitude, 2 s pulsed on/off). Afterward, solvents were vacuum spin evaporated for 15 min at 37 °C to completely remove the organic phase. Eventually, the solution was extruded 3 times through a polycarbonate membrane with a pore size of 200 nm to obtain liposomes.

#### Preparation of blank HENPs

For blank HENPs, HENPs were synthesized by membrane fusion technology. One hundred microliters of exosomes (2 mg mL^−1^) was mixed with 1 mg of liposomes in a final volume of 1 mL, vortexed and sonicated (33% amplitude, 2 s pulsed on/off, for 3 min) for proper mixing. Then, the mixture was vacuum vortexed for 15 min to completely remove the organic phase and finally extruded through a 200-nm polycarbonate membrane filter to obtain nanosized HENPs.

#### Synthesis of drug-loaded HENPs (miR497/TP-HENPs)

Liposomes first encapsulated TP and then underwent membrane fusion with exosomes. Next, a mixture of miR497 and a CaCl_2_ solution (100 μM) was added. Finally, HEPES was quickly added to the TP-HENPs/Ca^2+^/miR497 solution for 30 min at 4 °C to obtain miR497/TP-HENPs. The entrapment efficiency (EE%) of TP and miR497 in all samples was detected by high-performance liquid chromatography (HPLC, Agilent, USA) and fluorescence spectrophotometry (F2700, Hitachi, Japan), respectively.

#### Characterization of nanoplatforms

The surface morphologies of liposomes, exosomes and HENPs were observed by transmission electron microscopy (TEM, JEM-2100, Japan). The particle concentration and particle size of exosomes were detected by nanoparticle tracking analysis (NTA, Malvern, UK). The particle sizes of liposomes and HENPs and the surface potentials of liposomes, HENPs and exosomes were detected through a Zetasizer IV analyzer (Malvern, U.K.). Protein quantification of exosomes was performed by the BCA protein assay kit (Beyotime, Shanghai), and protein identification was characterized by the WB assay. Free miR497 and miR497-HENPs were incubated with 10% FBS for 2 h, 6 h, 24 h or 48 h, and then the distance traveled by miR497 was observed by a gel block test. Exosomes, liposomes and miR497/TP-HENPs were diluted with 10% FBS at 37 °C for 7 days to evaluate the storage stability by measuring the average size and polydispersity index (PDI).

#### Fluorescence resonance energy transfer study (FRET)

The FRET assay was performed to detect the fusion efficiency of liposomes and exosomes. FITC acting as an electron donor and RB acting as an electron acceptor were incorporated into the lipid mixture at a molar ratio of 1:1 (mol/mol), resulting in the formation of FRET liposomes. For fusion analysis, exosomes and FRET liposomes were mixed and ultrasonicated for 5 min at 4 °C to initiate fusion. FRET liposomes before and after fusion of exosomes were analyzed by fluorescence spectrophotometry (BioTek Instruments, USA) at an excitation wavelength of 488 nm, and the emission spectra were measured between 500 and 700 nm. The percentage of FRET efficiency was calculated by the following equation: % FRET efficiency = (I_RB_/(I_RB_ + I_FITC_)) × 100%, where I_RB_ = intensity of emitted fluorescence of the acceptor (RB) and I_FITC_ = intensity of emitted fluorescence of the donor (FITC).

#### TP and miR497 release study

The release pattern of miR497 from nanoparticles was detected by Cy5-labeled control miRNA (Cy5-miRNC). Cy5-miRNC-HENPs were dissolved in different pH solutions (5.5 and 7.4) and sealed in dialysis bags (MWCO = 100,000). These bags were placed into 10 mL of DEPC water in a centrifuge tube. Additionally, the equivalent concentration of free Cy5-miRNC was the control group. TP-HENPs were dissolved in solutions of different pH values (5.5 and 7.4), sealed in dialysis bags (MWCO = 3500) and placed in 10 mL of PBS solution. The equivalent concentration of free TP was the control group. All samples were placed in a shaker and shaken continuously at 37 °C at 90 rpm/min. At the scheduled point, 200 µl of solution was removed from each sample in a centrifuge tube, and the corresponding fresh solution was added. The fluorescence intensity of Cy5 and the quantity of TP were measured by fluorescence spectrophotometry and HPLC. Then, the release curves were plotted.

#### Cellular uptake of RB HENPs

Cellular uptake of rhodamine B (RB) HENPs was examined by confocal laser scanning microscopy (CLSM, Olympus, Japan, FV1000) and flow cytometry (FCM, Becton Dickinson, USA). SKOV3-CDDP and SKOV3 cells were spread in laser confocal dishes at a density of 5 × 10^4^ and cultured overnight. On the following day, cells were incubated with free RB, RB liposomes (RB Lipo) and RB HENPs (the concentration of RB was kept at 6 µg mL^−1^) and incubated for 2 h or 4 h. The cell nucleus was stained with Hoechst 33,342 (5 µg mL^−1^) for 10 min and then photographed under CLSM. Furthermore, after 4 h of treatment with different interventions, cells were harvested, and intracellular RB was quantified by FCM. Diverse internalization inhibitors were added to cells to block specific uptake routes. The fluorescence intensity of RB was quantified in different treatment groups with FCM.

#### CCK-8 assay

For determining the IC50 values, SKOV3-CDDP and SKOV3 cells were seeded at 5 × 10^3^ cells per well in 96-well plates and incubated overnight. After treatment with different drugs for 24 h or 48 h, 100 µl of DMEM containing 10% cell counting kit-8 (CCK-8, Yeasen, Shanghai, China) solution was added to each well and incubated for 1 h at 37 °C. Absorbance values were measured by an absorbance spectrophotometer at 450 nm.

#### Cytotoxicity of blank HENPs

SKOV3-CDDP cells and SKOV3 cells were seeded at a density of 5 × 10^3^ cells per well in 96-well plates. L929 cells were seeded at a density of 6 × 10^3^ cells, and RAW264.7 cells were plated at a density of 1 × 10^4^ cells per well in 96-well plates and cultured overnight. Blank HENPs were added at different concentrations for 24 h, 48 h and 72 h. Cell viability was determined by the CCK-8 assay.

#### Apoptosis

SKOV3-CDDP and SKOV3 cells were plated into 6-well plates at a density of 2 × 10^5^ cells overnight and treated with PBS, miR497, miR497-HENPs, TP, TP-HENPs and miR497/TP-HENPs for 48 h. Calcein-AM (10 nM) staining was applied for 10 min and photographed using fluorescence microscopy (Olympus IX51, Olympus Corporation, Japan). Cell apoptosis was also detected according to the instructions of the Annexin V FITC/PI double staining kit (Vazyme™, Nanjing). Briefly, the cells were washed three times, resuspended in 200 µl of binding buffer, combined with 5 µl of Annexin V FITC and PI, stained for 15 min at room temperature, protected from light, and subsequently analyzed by FCM.

#### Western blot

To examine the expression of the proteins in both cell lines, SKOV3-CDDP and SKOV3 cells were seeded at 2 × 10^5^ cells per well in 6-well plates and cultured overnight. The two cell lines were treated with PBS, miR497, miR497-HENPs, TP, TP-HENPs and miR497/TP-HENPs for 48 h. Then, 150 µl RIPA buffer (Sigma–Aldrich) was added and lysed on ice for 5 min. The lysate was collected and centrifuged at 12,000 g for 15 min at 4 °C. The protein content in the supernatant was quantified by a BCA protein assay kit. Protein samples were separated by SDS–PAGE and transferred to PVDF membranes, and the membranes were blocked with 5% blocking buffer for 1 h and then incubated overnight at 4 °C with the following primary antibodies: anti-calnexin, anti-TSG101, anti-CD9, anti-CD47, anti-GAPDH, anti-PI3K, anti-p-PI3K and anti-mTOR (purchased from Abcam) and anti-p-mTOR, anti-AKT, anti-p-AKT and HIF-α (obtained from CST). The membranes were washed three times, and then at room temperature, the secondary antibody was incubated for 1 h. All strips were visualized using a Bio–Rad Imaging System (Bio–Rad, USA).

#### GSH quantity and cellular ROS

SKOV3-CDDP cells were spread in 6-well culture plates and cultured overnight. The next day, different drugs were added and treated for 48 h. Finally, the total GSH in the cells was detected by a GSH assay kit according to the manufacturer’s instructions (Beyotime, Jiangsu). After adding 2,7-dichlorodihydrofluorescein-diacetate (DCFH-DA), fluorescence microscopy was used for imaging, and FCM was used to quantify the fluorescence intensity of 2,7-dichlorodihydrofluorescein (DCF), a detector of ROS.

#### Regulation of macrophage polarization

To investigate the regulatory effect of TP on macrophage repolarization, 5 × 10^5^ RAW264.7 macrophages were plated on six-well plates and cultured overnight. The following day, interleukin-4 (IL-4, 40 ng mL^−1^) was added to induce macrophage polarization to M2 macrophages. Then, the cells were treated with PBS, miR497, TP, TP-HENPs and miR497/TP-HENPs for 24 h. Finally, harvested cells were stained with zombie-apc-cy7, anti-F4/80-BV421, anti-CD86-PE and anti-CD206-APC, followed by quantitative analysis by FCM. ELISA was used to detect TNF-α and TGF-β1 concentrations in the cell supernatant according to the manufacturer’s instructions.

#### Establishment of mice bearing subcutaneous SKOV3-CDDP tumors

All animal experiments were performed in accordance with the guidelines and were evaluated and approved by the Committee of the Shanghai Cancer Institute. The mouse subcutaneous tumor model was used to assess the inhibitory effect of miR497/TP-HENPs on cisplatin-resistant OC. Healthy female 4- to 6-week-old BALB/c-nu mice weighing 18 ± 0.85 g were obtained from Shanghai Slack Laboratory Animals Co. Ltd. (Shanghai, China). Briefly, approximately 2 × 10^7^ SKOV3-CDDP cells were resuspended in 100 µl of PBS and injected subcutaneously into the right axilla of nude mice. Then, the tumor volumes of the mice were measured (the formula for calculating the volume of the tumor was V = a × b^2^ /2, where a represents the length and b is the width).

#### Targeting of Dir HENPs in vivo

When the subcutaneous tumor volume reached approximately 500 mm^3^, the fluorescent dye Dir (Yeasen, Shanghai, China) was loaded into nanoparticles to obtain Dir liposomes (Dir Lipo) and Dir HENPs. Free Dir, Dir Lipo and Dir HENPs (Dir, 0.5 mg kg^−1^) were injected into mice (n = 3) via the tail vein, and the distribution of Dir was observed by an in vivo imaging apparatus (Berthold Technologies, Germany) at preset time points (2 h, 4 h, 8 h, 24 h and 48 h). Forty-eight hours after injection, major organs and subcutaneous tumors were harvested and observed by an in vivo imaging apparatus.

#### In vivo anticancer effect

When the subcutaneous tumor volume reached approximately 100 mm^3^, the tumor-bearing mice were randomly divided into six experimental groups of six mice each. The corresponding treatments of PBS, miR497, miR497-HENPs, TP, TP-HENPs and miR497/TP-HENPs (TP, 0.2 mg kg^−1^, miR497, 250 nmol kg^−1^) were administered once every three days for one month via the tail vein, and the volume of tumors and body weight of the mice were measured before each administration. Before the mice were sacrificed, whole blood was collected, and liver and kidney function-related indicators were measured using biochemical assay kits. Additionally, the immune-related indicators TNF-α and TGF-β1 were detected by ELISA. Subcutaneous tumors were collected for hematoxylin and eosin (H&E) staining, terminal deoxynucleotidyl transferase dUTP nick-end labeling (TUNEL) assay, immunohistochemical (IHC) staining assay, ROS staining and macrophage polarization evaluation. The main organs (heart, liver, spleen, lungs and kidneys) were harvested for H&E staining.

#### Histology analysis of tumors

For H&E staining and TUNEL staining. OC tumors were fixed in formaldehyde solution, followed by staining with an H&E or TUNEL kit. All slices were observed and imaged by fluorescence microscopy.

For immunohistochemistry. Tumors were fixed in formaldehyde solution, and then sections were incubated with primary antibodies overnight at 4 °C. Next, the cells were washed with PBS and incubated with secondary antibodies. Finally, all slices were stained with hematoxylin and imaged by microscopy.

For ROS staining. Tumors were frozen at − 20 °C and fixed in acetone. Then, the tumor slices were incubated with DCFH-DA for 1 h and stained with DAPI for 10 min. All slices were imaged by fluorescence microscopy.

For the polarization of macrophages. Tumors were fixed and then incubated with primary antibodies against F4/80, CD86, and CD206 overnight at 4 °C, washed with PBS and incubated with secondary antibodies. Finally, the tumor slices were stained with DAPI, and images were captured by fluorescence microscopy.

#### Statistical analysis

Experimental data are presented as the mean ± standard deviation (mean ± SD) and were analyzed by the independent t test for comparisons between two groups or one-way analysis of variance (ANOVA) for comparisons among three or more groups with GraphPad Prism software 8.0. p < 0.05 was considered to be a statistically significant difference (ns: p > 0.05, *p < 0.05, **p < 0.01, ***p < 0.001).

## Supplementary Information

**Additional file 1:** **Figure S1.** ^1^H NMR spectra were obtained to confirm the successful synthesis of DSPE-PEG_1k_-cRGD in CDCl3. **Figure S2.** Representative images of liposomes captured by TEM at different magnifications. **Figure S3.** The efficiency of the fluorescence resonance energy transfer (FRET). **Figure S4.** Cell viability of four cell lines treated with blank HENPs. **Figure S5.** A gel blocking assay was performed to detect the protective function of miR497 by miR497-HENPs. **Figure S6.** Cellular uptake of HENPs in vitro. **Figure S7.** CD47 on the exosome surface avoided nanoparticle clearance by the MPS system. **Figure S8.** In vitro toxicity of cisplatin and triptolide. **Figure S9.** Cell viability of SKOV3-CDDP and SKOV3 cells with various treatments. **Figure S10.** Absorbance values at 450 nm of SKOV3-CDDP and SKOV3 cells with various treatments at pH 5.5. **Figure S11.** Quantification of the fluorescence intensity of calcein-AM staining.

## Data Availability

All data and materials about this study are included in this published article and its additional files.
