# Supplementary material for: Exosome-liposome hybrid nanoparticle codelivery of TP and miR497 conspicuously overcomes chemoresistant ovarian cancer
Source: J Nanobiotechnology. 2022 Jan 25;20:50. doi: 10.1186/s12951-022-01264-5 (PMC8787930; doi:10.1186/s12951-022-01264-5)
Supplement: Supplementary file 1 — Additional file 1: Figure S1. 1H NMR spectra were obtained to confirm the successful synthesis of DSPE-PEG1k-cRGD in CDCl3. Figure S2. Representative images of liposomes captured by TEM at different magnifications. Figure S3. The efficiency of the fluorescence resonance energy transfer (FRET). Figure S4. Cell viability of four cell lines treated with blank HENPs. Figure S5. A gel blocking assay was performed to detect the protective function of miR497 by miR497-HENPs. Figure S6. Cellular uptake of HENPs in vitro. Figure S7. CD47 on the exosome surface avoided nanoparticle clearance by the MPS system. Figure S8. In vitro toxicity of cisplatin and triptolide. Figure S9. Cell viability of SKOV3-CDDP and SKOV3 cells with various treatments. Figure S10. Absorbance values at 450 nm of SKOV3-CDDP and SKOV3 cells with various treatments at pH 5.5. Figure S11. Quantification of the fluorescence intensity of calcein-AM staining. [file 12951_2022_1264_MOESM1_ESM.docx]

**Additional information**

**Exosome-Liposome Hybrid Nanoparticle Codelivery of TP and miR497 Conspicuously Overcomes Chemoresistant Ovarian Cancer**

Longxia Li^a^, Di He^a^, Qianqian Guo^b^, Zhiyoung Zhang^c^, Dan Ru^a^, Liting Wang^b^, Ke Gong^b^, Fangfang Liu^a^, Yourong Duan^b,^ * and He Li^a,^ *

^a^ Traditional Chinese Medicine Department, Renji Hospital, School of Medicine, Shanghai Jiao Tong University, Shanghai 200127, China

^b^ State Key Laboratory of Oncogenes and Related Genes, Shanghai Cancer Institute, Renji Hospital, School of Medicine, Shanghai Jiao Tong University, Shanghai, China

^c^ Huashan Hospital and Key Laboratory of Medical Epigenetics and Metabolism and Molecular and Cell Biology Lab, Institute of Biomedical Sciences, Shanghai Medical College, Fudan University, Shanghai 200032, China.

*Corresponding authors: He Li; E-mail: [lihe1972@hotmail.com](mailto:lihe1972@hotmail.com)

Yourong Duan; E-mail: [yrduan@shsci.org](mailto:yrduan@shsci.org)


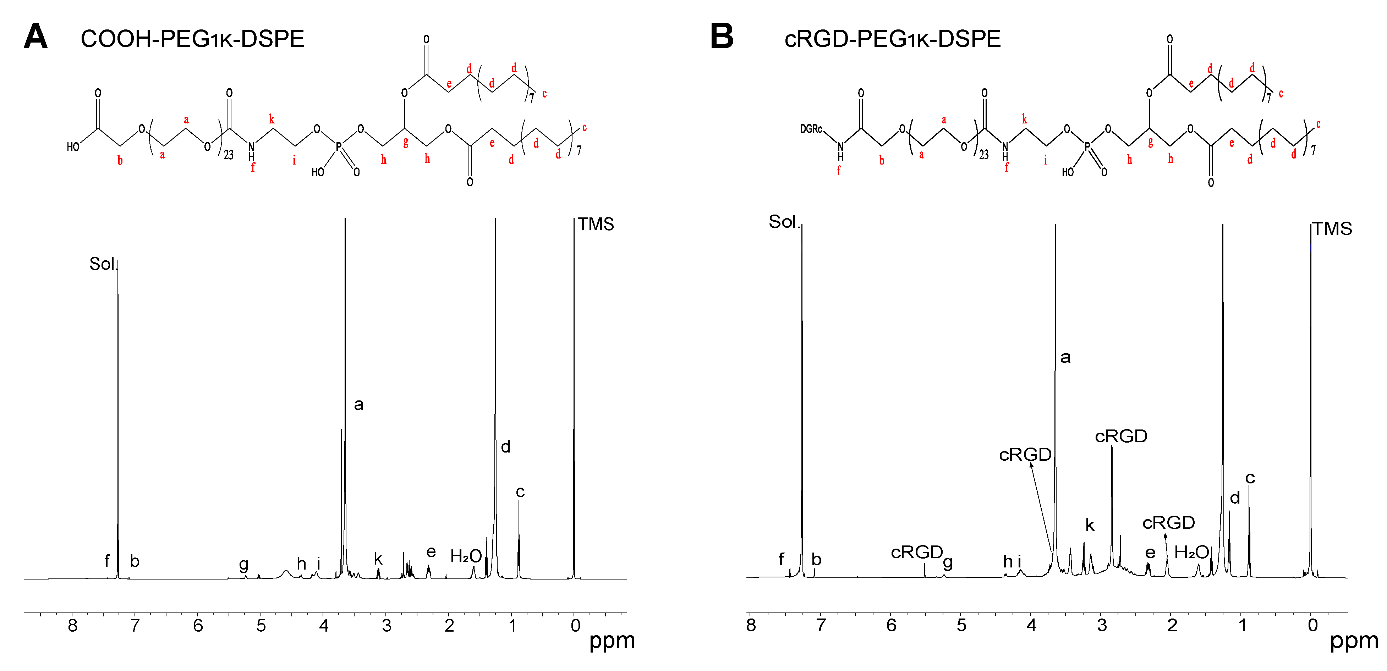


**Figure S1.** The ^1^H NMR spectra was performed to confirm the successful synthesis of the DSPE-PEG_1k_-cRGD in CDCl_3_. (A) DSPE-PEG_1k_-COOH and (B) DSPE-PEG_1k_-cRGD. The characteristic peaks were pointed out.


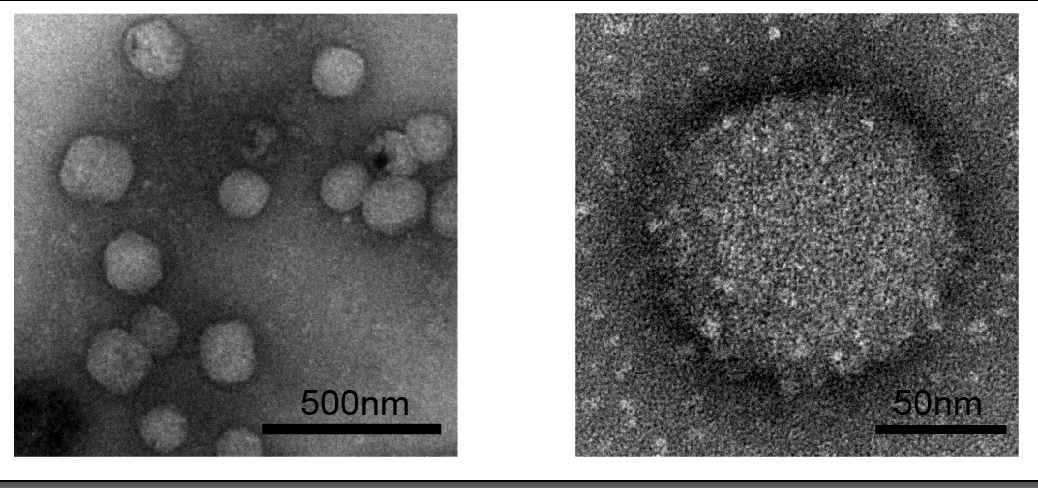


**Figure S2.** Representative images of liposomes captured by TEM at different magnifications.


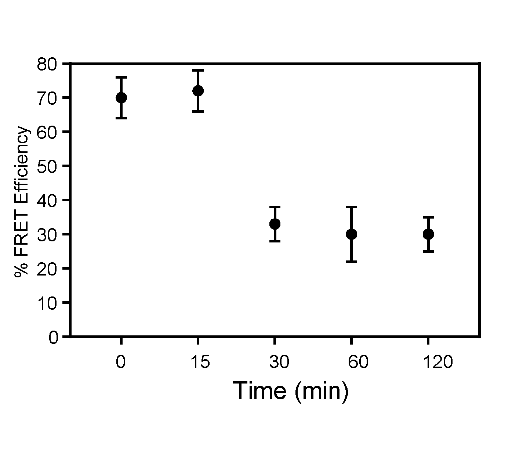


**Figure S3.** The efficiency of fluorescence resonance energy transfer (FRET).


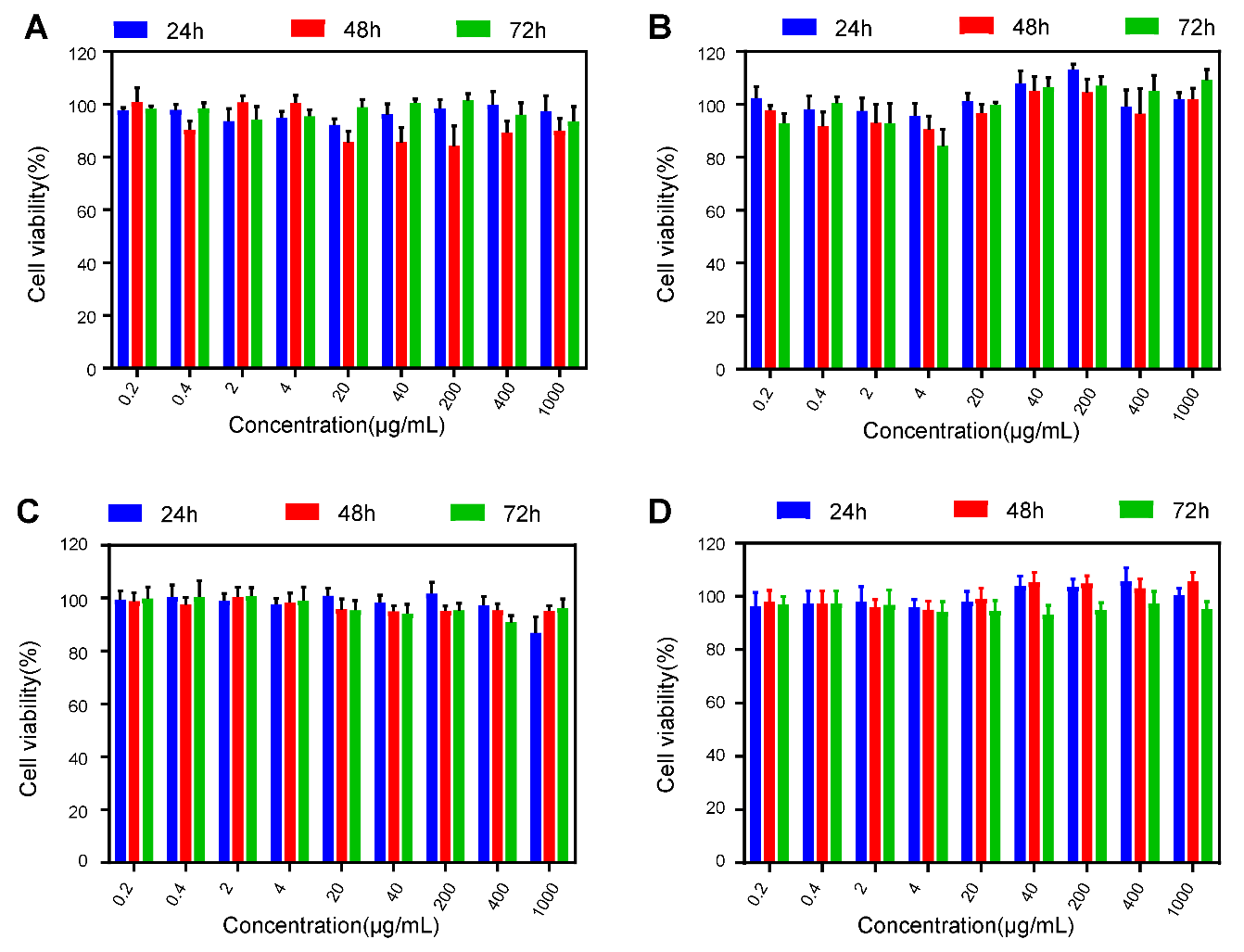


**Figure S4.** Cell viability of four cells were treated with blank HENPs. (A)SKOV3-CDDP. (B)SKOV3. (C) L929 cells. (D) RAW264.7 cells. There was no significant decrease in the viability of four cells with incubation with different concentration of blank HENPs for 24 h, 48 h and 72 h.


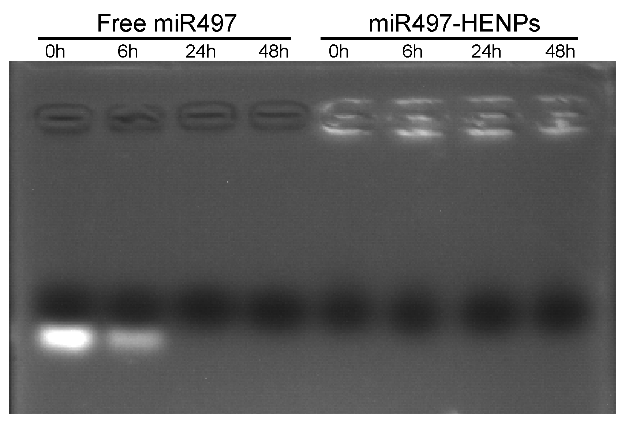


**Figure S5.** Gel blocking assay was performed to detect the protective function of miR497 by miR497-HENPs.


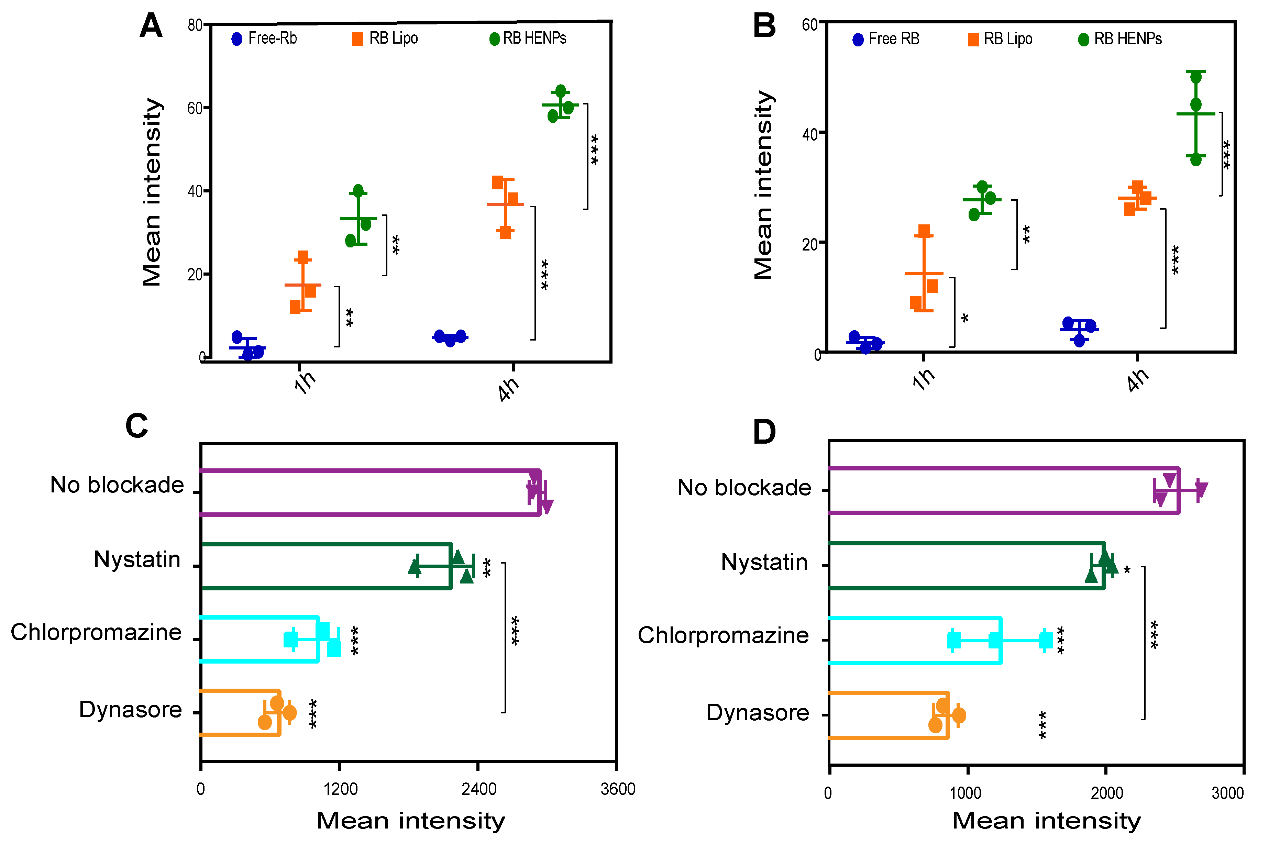


**Figure S6.** Cellular uptake of HENPs in vitro. (A, B) Quantitative fluorescence intensity of RB in SKOV3-CDDP and SKOV3 cells. (C, D) Quantitative fluorescence intensity of RB after the addition of various uptake inhibitors in SKOV3-CDDP and SKOV3 cells.


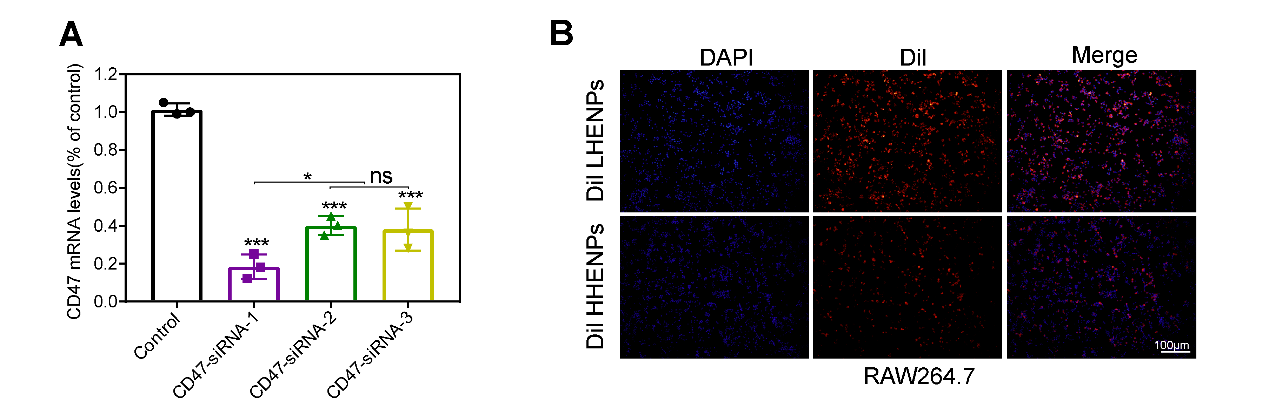


**Figure S7.** CD47 on the exosome surface avoided nanoparticle clearance by the MPS system. (A)Gene knockdown efficiency of CD47 siRNA. (B) Cellular uptake of Dil LHENPs and Dil HHENPs in vitro.


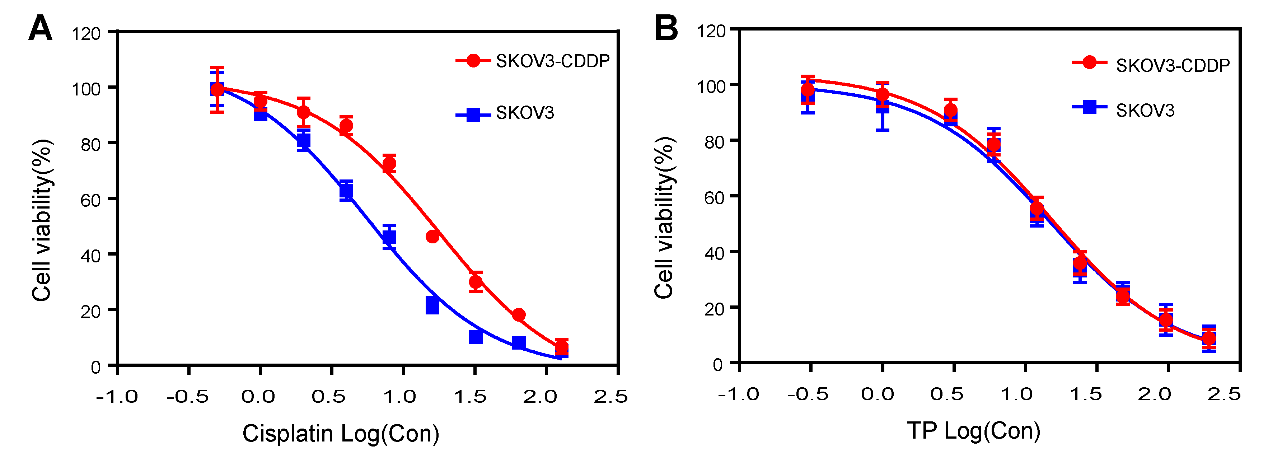


**Figure S8.** In vitro toxicity of Cisplatin and Triptolide. Cell viability of SKOV3-CDDP cells and SKOV3 cells treated with different concentrations of Cisplatin (A) and TP (B) in 48 h.


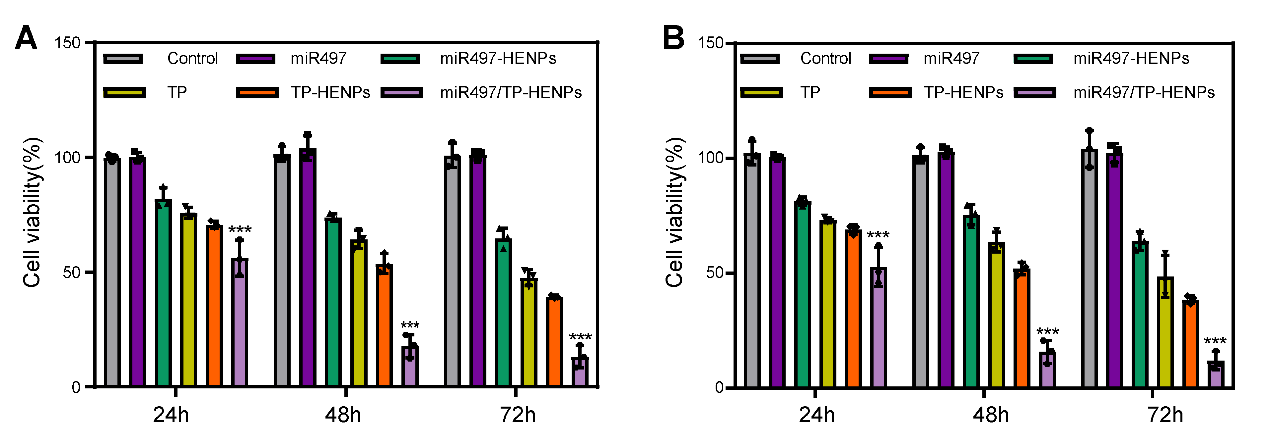


**Figure S9.** Cell viability of (A) SKOV3-CDDP and (B) SKOV3 cells with various treatments for 24 h, 48 h and 72 h by CCK8 assay.


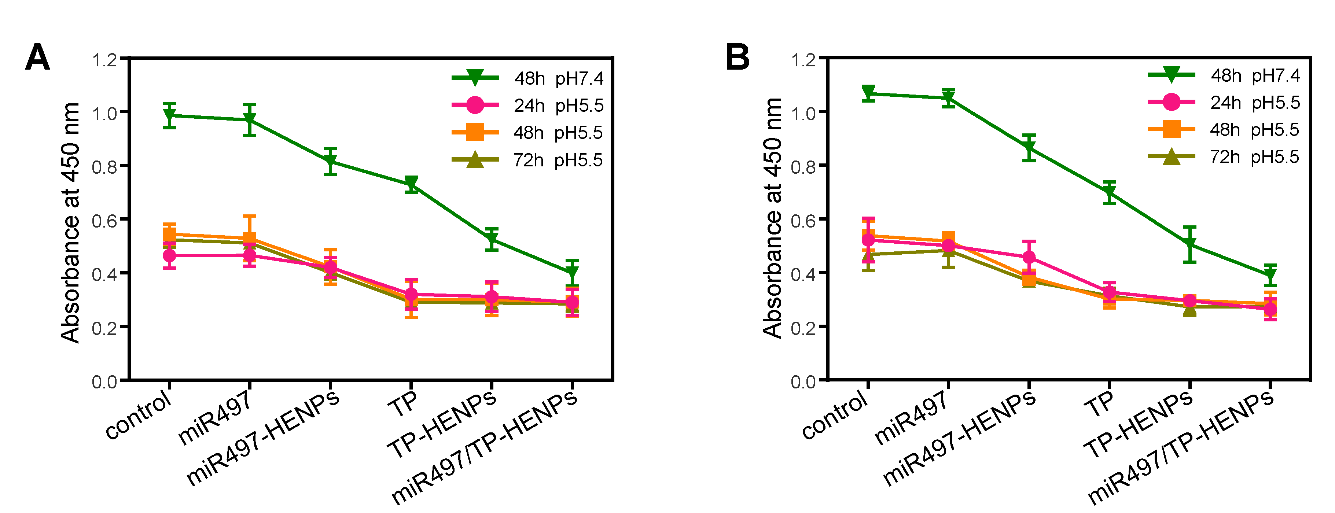


**Figure S10.** Absorbance values at 450 nm of (A) SKOV3-CDDP and (B) SKOV3 cells with various treatments for 24 h, 48 h and 72 h by CCK8 assay at pH 5.5.


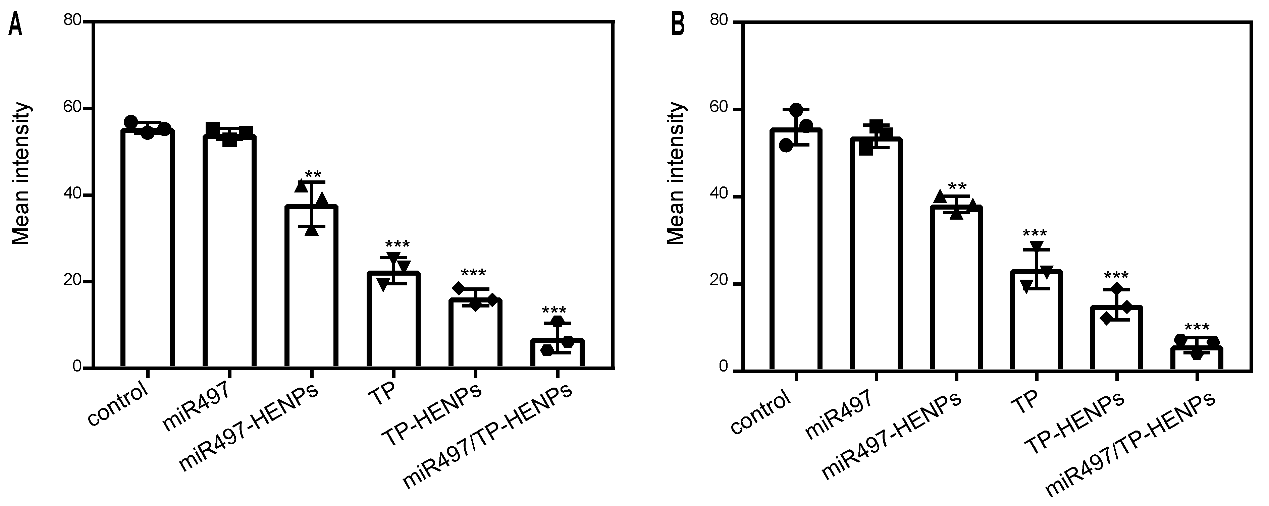


**Figure S11.** Quantification of fluorescence intensity of calcein-AM staining of (A) SKOV3-CDDP and (B) SKOV3.­
